# Dual Branding by National Brand Manufacturers: Drivers and Outcomes

**DOI:** 10.1177/00222429231196575

**Published:** 2023-11-15

**Authors:** Yu Ma, Kusum L. Ailawadi, Mercedes Martos-Partal, Óscar González-Benito

**Keywords:** private label, private label supply, dual branding, national brands, distribution depth

## Abstract

This article is the first generalizable empirical analysis of dual branding, that is, the supply of private labels (PLs) by national brand (NB) manufacturers. The authors compile a unique data set combining the identity of PL suppliers in over 260 packaged goods categories with multiple years of scanner data in the Spanish grocery market to offer several contributions. First, they provide new descriptive insights on the prevalence of dual branding in categories where the manufacturer does and does not have NBs, the longevity of PL supply arrangements, and the differences in PL sourcing across retailers. Second, they integrate the literature on motivators and dissuaders of dual branding and test the impact of relevant manufacturer, retailer, and dyad characteristics on PL supply in NB and non-NB categories. The results reveal a more nuanced empirical reality than is evident from prior research regarding the role of multicategory scope, fighter brands, NB differentiation, and size and positioning of the retailer's PL. Third, they examine the outcomes of PL supply for the NBs of dual branders and find that starting (terminating) PL supply to a retailer significantly benefits (hurts) the relative distribution depth but not the relative share of the dual brander's NBs at that retailer.

Private labels (PLs) form a substantial portion of consumer packaged goods (CPG) sales in many countries and receive a great deal of attention from practitioners and academics alike. There is a large body of empirical work examining various aspects of consumer response to PLs (e.g., Erdem, Zhao, and Valenzuela 2004; Geyskens, Gielens, and Gijsbrechts 2010; Keller, Dekimpe, and Geyskens 2016; Sethuraman and Gielens 2014; Szymanowski and Gijsbrechts 2012) and the effect of PLs on manufacturers and/or retailers (e.g., Ailawadi and Harlam 2004; Chintagunta, Bonfer, and Song 2002; Pauwels and Srinivasan 2004).

When it comes to the supply side of PLs, there is a real dearth of empirical research despite an abundance of conceptual and theoretical work. It is an open secret that national brand (NB) manufacturers also supply PLs in what is referred to as dual branding. Several conceptual articles (e.g., Dunne and Narasimhan 1999; Hoch 1996; Quelch and Harding 1996) and books (e.g., De Jong 2019; Kumar and Steenkamp 2007; Steenkamp and Sloot 2019) have discussed the pros and cons of dual branding. Many of them cite Conagra, Kimberly-Clark, and R.J. Reynolds as examples of NB manufacturers that supply PLs. A common theme in this writing is that PL supply offers a revenue source, especially for secondary brands, but it risks the health of the manufacturers’ NBs, and profitability can be challenging for any but the most efficient producers.

This conceptual work has served as a point of departure for several analytical models that explain why dual branding may make sense despite the downside (e.g., Amaldoss and Shin 2015; Kumar, Radhakrishnan, and Rao 2010; Nasser, Turcic, and Narasimhan 2013; Soberman and Parker 2006). These models provide rationales such as price discrimination, channel coordination, and category management for why it may be optimal for a NB manufacturer to supply PL goods to a retailer and for a retailer to source its PL from a NB manufacturer.

However, little is known about the types of NB manufacturers that actually supply PLs in different categories to different retailers, whether the implications of analytical models hold up in the marketplace, and whether NBs of dual branders are affected at the retailers for which they supply PLs. While it is an open secret that dual branding occurs, which manufacturers supply PLs to which retailers is generally a well-kept one. In fact, it is not even clear how prevalent dual branding is. At one end, Quelch and Harding (1996) note, without providing a source, that more than 50% of NB manufacturers supply PLs. At the other end, Hoch (1996) notes that about 5% of the PL suppliers listed in the PL Product News *Supplier Source Book* were NB manufacturers, most of them secondary brands.

Further, prior work implicitly or explicitly considers dual branding in categories where manufacturers already have NBs. But there is no reason to believe that PLs are not, or should not be, supplied by a dual brander outside its NB categories (hereinafter “non-NB categories”). After all, doing so offers manufacturers an opportunity to earn revenue without competing with their own NBs. Of course, the aforementioned theoretical motivations for PL supply diminish without a NB in the category that can benefit from coordination and category management. Retailers may also be wary of sourcing a PL from a supplier that does not have experience in the category. How these considerations trade off, which types of dual branders supply PLs in non-NB categories to which retailers, and how frequently they do so are unknown.

In sum, most of what we know about dual branding, its antecedents, and its consequences is anecdotal and/or theoretical. And even the theoretical knowledge does not extend to PL supply beyond the manufacturer's NB categories. An important empirical exception is the work of Ter Braak, Deleersnyder, et al. (2013), who study PL supply by leading NB manufacturers (specifically, the largest five in a category) at one PL-focused discount retailer each in Spain and Germany. Both retailers had begun to stock a few NBs in some of their categories at the time of the authors’ data. In answering their key research question, which is whether supplying PLs to these retailers is associated with a higher probability of the manufacturers’ NBs in the category being stocked by the retailer (they find that it is), these authors also model the PL supply decision as a function of mainly manufacturer characteristics. This work is both novel and important. However, it is unclear whether the findings can be generalized or are unique to the specific context of leading NB manufacturers supplying PLs in their NB categories to PL-focused discounters.

For example, these discounters have, by definition, a large PL business that may attract, indeed necessitate, large suppliers while their discount positioning may deter manufacturers whose expertise is in developing premium products. However, most other retailers stock a large assortment of NBs in addition to a PL, and they vary in the size and positioning of the PL as well as in the prominence of various NBs on their shelves. As a result, not only may manufacturer characteristics play different roles in driving the PL supply decision, but other aspects such as the size and differentiation of the retailer's PL and characteristics of the specific manufacturer–retailer dyad also come into play. Furthermore, manufacturers whose NBs are already stocked by a retailer may or may not see a goodwill boost in the depth of their distribution due to PL supply. It is possible, for example, that PL supply is a reciprocal action that the retailer expects from NB manufacturers that are already prominent in its stores. It is also unclear whether a boost in distribution, if any, will be accompanied by a boost in share. After all, stocking more of the manufacturer's products is under the control of the retailer, but generating more sales depends on consumer preferences. Finally, Ter Braak, Deleersnyder, et al. (2013) do not examine the possibility that a dual brander may supply PLs in its non-NB categories, so we do not know how prevalent that practice is or what its drivers are.

These issues, and the scarcity of empirical work on the prevalence, antecedents, and consequences of PL supply by NB manufacturers, motivate our research. Our objective is to conduct a comprehensive and generalizable empirical analysis of dual branding by (1) documenting the incidence and patterns of PL supply by NB manufacturers across retailers; (2) identifying and testing the theory-based manufacturer, retailer, and dyad drivers of PL supply by NB manufacturers and investigating how their roles differ in the manufacturers’ NB and non-NB categories; and (3) examining the distribution depth and share consequences for the NBs of dual branders at retailers to which they begin or stop supplying PLs.

We accomplish this with unique data. For six of the largest retailers in the Spanish grocery market, we obtain the identities of PL suppliers in over 260 categories in two years (2012 and 2017). We combine this information on PL suppliers with longitudinal home-scan purchase data in the market before and during that period (from 2008 to 2017), spanning almost 3,500 NB manufacturers and, of course, PLs. The six retailers account for about 75% of tracked CPG purchases and vary in their price positioning, PL emphasis, and market shares. Our research focuses on findings that generalize across retailers, and we preview a few of them subsequently.

More than 70% of the PL suppliers across these retailers are dual branders, and almost 30% of their PL supply arrangements are in non-NB categories. In NB categories, the two most important drivers of PL supply are the size of the manufacturer and its dependence on the retailer. In an interesting juxtaposition, both are positively associated with PL supply. Contrary to received wisdom, a PL does not seem to be a substitute for a fighter brand (fighter brands are launched by premium brand manufacturers to combat low-priced competitors). And differentiated NB manufacturers do not refrain from supplying PLs, especially when the retailer's PL is priced closer to NBs. Consistent with received wisdom about the quest for category influence and goodwill, NB manufacturers are more likely to supply a PL to a retailer when they face intense competition with other NBs and have lower distribution depth on the retailer's shelf. In non-NB categories, multicategory NB experience is the biggest driver of PL supply. However, several other characteristics, including the size and growth of the manufacturer's NBs, also play a role. Finally, manufacturers that enter into a PL supply arrangement with a retailer in their NB categories see a significant and substantial increase in their NB distribution depth at that retailer relative to the market. Conversely, there is a substantial decrease for manufacturers that exit a PL supply arrangement. Yet, there is no significant impact of either entry or exit on the share of category sales that the manufacturer's NBs get at the retailer relative to the market.

The rest of the article is organized as follows. In the next section, we briefly review the relevant literature and develop our conceptual framework. Next, we present our data and the descriptive patterns of PL supply to the different retailers in the market. The subsequent two sections tackle our analysis of PL supply in NB and non-NB categories (Study 1) and our analysis of the distribution and share outcomes for NBs of dual branders at retailers where they enter or exit PL supply arrangements (Study 2). We conclude with a discussion of our findings and implications for managers and researchers.

## Conceptual Development

To say that PLs are perceived by many NB manufacturers as the bane of their existence would not be an overstatement. PLs compete with them for the consumer's share of wallet, are generally priced significantly lower, are increasingly of comparable quality, and are given preference by retailers whose percentage gross margins are often higher on PLs than on NBs. Yet, NB manufacturers are often the ones that supply PLs to retailers.

**Figure 1.**
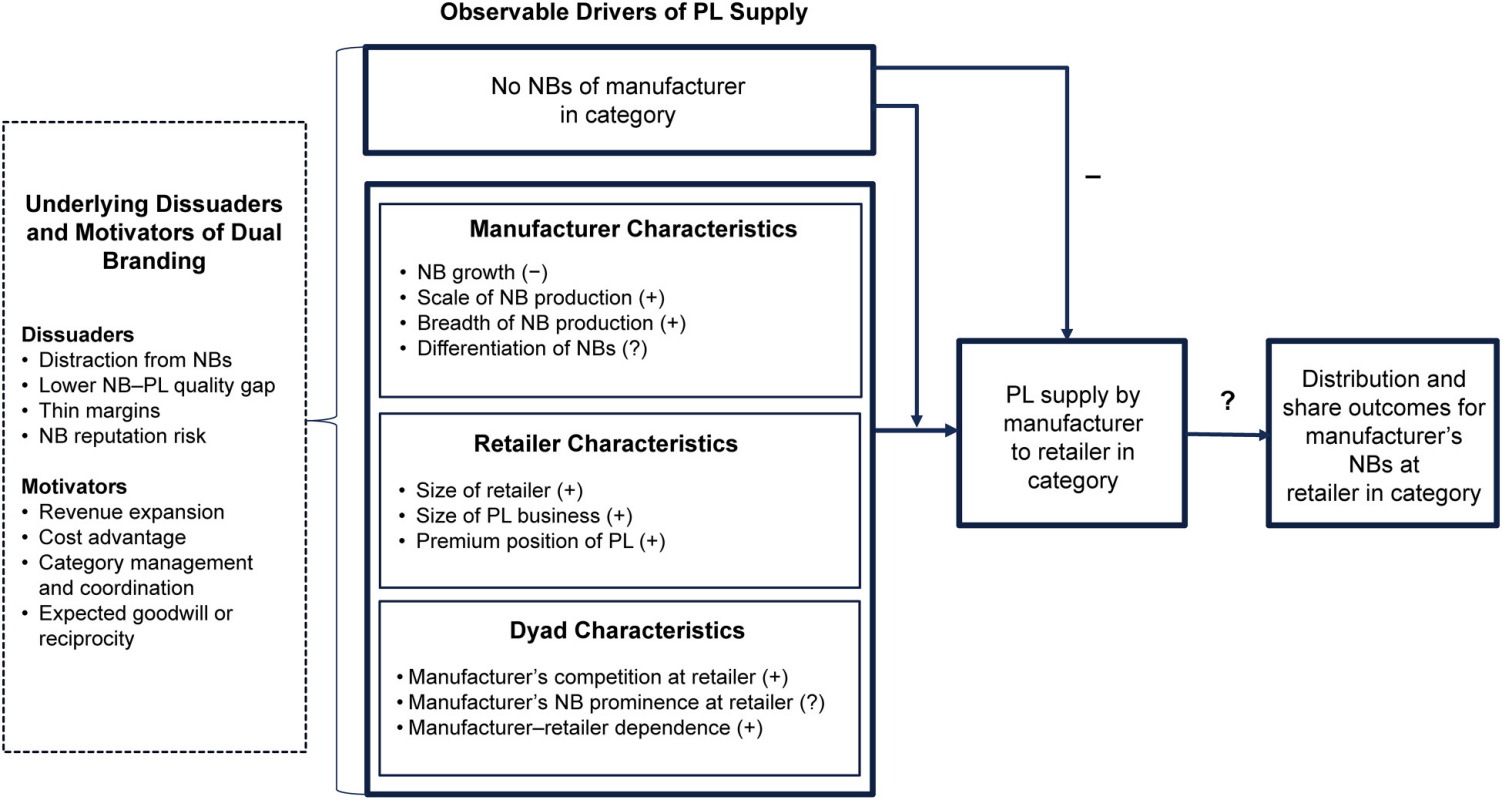
Drivers and Outcomes of PL Supply.

*Notes*: Expected main effects are shown. Moderator effects are discussed in the text.

We begin our conceptual development (summarized in Figure 1) with an overview of the potential dissuaders and motivators of dual branding and discuss how these may be less or more pronounced in non-NB versus NB categories. Next, we identify observable characteristics of the manufacturer, the retailer, and the manufacturer–retailer dyad that may strengthen or weaken these underlying dissuaders and motivators and therefore may drive each party's willingness and/or ability to enter into a PL supply arrangement in a particular category. These steps guide Study 1 of our subsequent empirical analysis. We then close the loop by discussing how, if at all, PL supply in a category in which the manufacturer has NBs may affect the distribution and share outcomes of those NBs at the retailer. This step guides Study 2 of our empirical analysis.

### Dual Branding: Underlying Dissuaders and Motivators

Several factors dissuade NB manufacturers from supplying PLs. It can distract them from their brand-building efforts (Nasser, Turcic, and Narasimhan 2013; Quelch and Harding 1996). It enables retailers to have too much visibility into their innovations and costs, and the retailers’ demands can shrink the gap between the quality and image of their own NBs and the PL (Steenkamp, Van Heerde, and Geykens 2010), thus feeding what Kumar and Steenkamp (2007, p. 141) refer to as the “vicious circle of dual strategy.” This is especially problematic because PLs have very thin margins, so much so that researchers often assume perfect competition in PL supply (Chen et al. 2010; Nasser, Turcic, and Narasimhan 2013). There is also a risk to the reputation of dual branders’ NBs if consumers become aware that they manufacture PLs in addition to NBs and question whether the NBs are superior in quality (Hoch 1996).

Despite these dissuaders, why do at least some NB manufacturers supply PLs? From the retailers’ perspective, it is desirable to have PL suppliers with expertise in the category to provide PL quality on par with that of NBs. Hence, retailers that have some leverage may convince NB manufacturers to supply their PL. But also some potential benefits of PL supply may motivate NB manufacturers. One motivation, of course, is that PLs are a source of additional revenue. Why not try to take a piece of the PL pie instead of leaving it to the competition (Dunne and Narasimhan 1999)? This may be especially appealing if the manufacturer's NBs face slowing growth and if its production experience provides a cost advantage. Cost advantages have been front and center in most discussions of when NB manufacturers should supply PLs (Dunne and Narasimhan 1999; Kumar and Steenkamp 2007; Mills 1999; Nasser, Turcic, and Narasimhan 2013; Peles 1972; Quelch and Harding 1996; Steenkamp and Sloot 2019).

Another motivator is the quest for category influence and coordination. NB manufacturers may be able to gain influence in a retailer's category management by supplying its PL (Chen et al. 2010; Dunne and Narasimhan 1999; Gomez-Arias and Bello-Acebron 2008; Kumar, Radhakrishnan, and Rao 2010). Ultimately, the retailer makes the pricing and promotion decisions in its stores, but it too can benefit from the coordination when it sources its PL from a NB manufacturer (Amaldoss and Shin 2015).

A final motivator is the manufacturers’ hope that supplying a retailer's PL will generate goodwill for their NBs from the retailer (Kumar and Steenkamp 2007; Ter Braak, Deleersnyder, et al. 2013), or, conversely, the retailer's expectation that a NB manufacturer that is doing well in its stores will reciprocate by supplying the retailer's PL. These dissuaders and motivators are shown on the far left in Figure 1. The dotted box signifies that they are not directly observed.

### How Dissuaders and Motivators Change Without a NB in the Category

How do these considerations for PL supply change when the manufacturer does not have NBs in the category? From the manufacturer's perspective, supplying a PL in a non-NB category offers revenue while limiting some of the risk to its NBs: direct competition is curtailed, as are the negative consequences for the NBs if the manufacturer's identity as a PL supplier becomes known. Also, retailers do not get as much of a window into NB operations whence to apply pressure to reduce NB prices or incorporate NB innovations into the PL.

However, the potential motivators of PL supply are also curtailed, perhaps more so. Manufacturers have less expertise in non-NB categories, and cost efficiencies are likely to be lower without learning curve advantages. The opportunity for NB and PL coordination is foregone, and goodwill benefits to NBs are reduced. The more different a category is from the ones in which the manufacturer has NB experience, the more the potential motivators of PL supply are curtailed. For their part, retailers also value category expertise, production efficiencies, and coordination benefits. Thus, they may be leery of sourcing a PL from a manufacturer without NB experience in the category. Therefore, despite PL supply creating direct competition against manufacturers’ own NBs, we expect that NB manufacturers will be more likely to supply PLs in their NB categories than in non-NB categories (top box in the middle of Figure 1).

Next, we discuss how observable manufacturer, retailer, and dyad characteristics may strengthen or weaken the underlying dissuaders and motivators of dual branding and therefore drive PL supply (remaining boxes in the middle of Figure 1). Throughout our discussion, we continue to incorporate the perspective of both manufacturers and retailers since a PL supply arrangement only occurs if both parties agree.

### Observable Manufacturer Characteristics

#### NB growth

Manufacturers whose NBs are not growing will be more willing to supply PLs as an alternative source of revenue and to avoid the cost of unused capacity. Indeed, it has often been said that dual branders tend to be secondary or fringe brands looking for additional revenue (Hoch 1996; Kumar and Steenkamp 2007; Quelch and Harding 1996). Retailers may, for their part, be able to negotiate favorable PL terms with such manufacturers and be more willing to source from them as reliable suppliers with available capacity. Growing manufacturers, in contrast, would not want to devote scarce capacity to PLs versus their own NBs; nor would retailers view them as reliable PL sources. Therefore, we expect NB manufacturers whose NBs are not growing to be more likely to supply PLs in their NB categories. Such manufacturers may also seek PL revenue in non-NB categories, though we expect the association to be weaker given the lack of experience and expertise in such categories.

#### Scale of NB production

NB manufacturers that have greater scale of NB production experience in a category are more able to supply PLs because of the ensuing economies and cost advantage. They may also be more willing to do so because scale economies enable reasonable margins despite the low manufacturer selling price of PLs. Retailers, for their part, look for a reliable and efficient PL supply at low prices (e.g., De Jong 2019; Deloitte 2015), so they are more willing to source from such NB manufacturers. Because scale advantages gained from NB production may not easily stretch beyond those categories, we expect the positive association to be weaker in non-NB categories.

#### Breadth of NB production

Manufacturers with broad NB production experience within and across categories may be able to cost-effectively satisfy the unique PL demands of retailers. Retailers seek differentiated PLs that can garner consumer loyalty to their stores (Ailawadi, Pauwels, and Steenkamp 2008; González-Benito and Martos-Partal 2012) and have their own requirements with respect to the formulation, packaging, and so on of their PLs (De Jong 2019; Steenkamp and Sloot 2019). That is why we expect manufacturers that produce more NBs in more categories to be more likely to also supply PLs in those categories. Furthermore, such manufacturers should be able to deploy their diverse capabilities to produce PLs in more non-NB categories than manufacturers with narrower scope could produce. From the retailer's perspective, broad scope of production should be especially important if the supplier does not have NB experience in the focal category. Therefore, we expect the association between scope of production and PL supply to be stronger in non-NB categories.

#### Differentiation of NBs

The dissuaders of dual branding summarized previously are likely to be especially salient for manufacturers that have differentiated their NBs. They do not want to endanger the equity of their NBs and reduce consumers’ willingness to pay a premium price for those brands. Theoretical work also suggests that the strategic gain from channel coordination, which we discuss under dyad characteristics, may be less useful for a differentiated NB because it is less substitutable with PLs (Chen et al. 2010). These arguments suggest that manufacturers of differentiated NBs should be less willing to supply PLs in their NB categories even though retailers want to benefit from their quality and innovativeness (Kumar and Steenkamp 2007).

However, some motivators are also likely to be more salient for differentiated NBs. Supplying a PL offers a premium NB manufacturer the opportunity to expand revenue at the low-price end of the market (Hoch 1996). This is an alternative to introducing a fighter NB of its own, especially if it is difficult to differentiate a longer NB product line (Nasser, Turcic, and Narasimhan 2013). Indeed, supplying a PL may be a profitable price discrimination strategy whereby the NB manufacturer targets its higher-priced and advertised NB to the quality-sensitive segment and the PL to the price-sensitive segment (Soberman and Parker 2006). Empirically, Ter Braak, Deleersnyder, et al. (2013) document a negative association between differentiation (as measured by the NB's price premium) and PL supply for one hard discounter. Given the limited empirical evidence and the arguments on both sides, we do not have a directional expectation about whether manufacturers of differentiated NBs would be more or less likely to supply PLs in their NB categories. In non-NB categories, the dissuaders as well as the motivators become weaker. The risk to NBs is lower, as noted previously, and the point about fighter brands and price discrimination becomes moot without a NB in the category. Therefore, we expect a weaker association of the manufacturer's NB differentiation with PL supply in its non-NB categories.

### Observable Retailer Characteristics

If NB manufacturers are wary of getting into the PL business, a retailer may have to exert some leverage and/or lure them.

#### Size of retailer

A large retailer should be able to more easily convince NB manufacturers to supply its PL while also negotiating attractive supply terms (Ter Braak, Dekimpe, and Geyskens 2013). After all, there is more to lose from an unhappy retailer if it has a substantial share of the category. We expect the positive association between retailer size and PL supply to be weaker in non-NB categories as large retailers should be less likely to press manufacturers for PL supply if they do not have NB experience.

#### Size of retailer's PL business

Since revenue expansion is a motivator for PL supply, NB manufacturers should be more willing to supply a PL to a retailer in a category if the retailer has a large PL business in the category. Any fixed costs that it incurs to meet the unique needs of a retailer are also more likely to be recuperated with a large sales base. This should also be a factor in non-NB categories, though we expect it to be weaker due to the manufacturer's lack of experience.

#### Premium position of retailer's PL

There is a greater opportunity to earn a reasonable margin by supplying the retailer's PL if the PL has a premium position (Ter Braak, Dekimpe, and Geyskens 2013), so NB manufacturers should be more willing to supply it. We expect this to also be attractive when manufacturers consider PL supply in non-NB categories, but, again, they are likely to be weaker draws because the manufacturer does not have NB experience in the category.

### Observable Dyad Characteristics

#### Manufacturer's competition at retailer

The category management and coordination benefits of PL supply are likely to be more valuable when competitive intensity in the category is high at the retailer because many more moving parts must be managed and coordinated. Thus, we expect a positive association with PL supply when a manufacturer's NBs face more competitors in the category on the retailer's shelf. These benefits are not available if the manufacturer does not have a NB in a category. However, as competition intensifies for their NBs, manufacturers may be more likely to seek revenue elsewhere. Hence we expect competitive intensity to be positively associated with PL supply in non-NB categories as well, albeit less strongly than in NB categories.

#### Manufacturer's NB prominence at retailer

Manufacturers whose NBs have room to improve their position at the retailer may be more willing to supply PLs in the hope of generating goodwill for themselves (Kumar and Steenkamp 2007; Ter Braak, Deleersnyder, et al. 2013). Those that are already prominent at the retailer (e.g., with higher distribution depth and/or higher share of the retailer's category sales) may have less need or room for improvement. Also, they may already be entrusted with category captain roles or otherwise have influence on category management. However, a retailer may expect such NB manufacturers to reciprocate by supplying PLs. Retailers may also be more willing to source PLs from manufacturers whose NBs have demonstrated high demand from their clientele. Thus, between the manufacturer's quest for goodwill and the retailer's expectation of reciprocity, it is unclear whether the prominence of a manufacturer's NBs at a retailer would be positively or negatively associated with PL supply. Finally, a manufacturer may expect goodwill in a NB category from supplying a PL in a non-NB category, and a retailer may expect reciprocity in a non-NB category from a manufacturer whose NBs are prominent in its stores. Both spillover expectations are likely to be weaker. Therefore, the association with PL supply in non-NB categories is also unclear.

#### Manufacturer–retailer dependence

More dependent parties in a channel dyad are subject to more pressure from their partners, have more to gain from maintaining the relationship, and/or have more to lose from its termination (Scheer, Miao, and Palmatier 2015). The more dependent the NB manufacturer is on a retailer, the more willing it should be to supply PLs to that retailer, both because of the pressure to accede to the retailer's request and to gain goodwill benefits. A retailer may also be more willing to source PLs from a NB manufacturer that is more dependent on it and hence more likely to prioritize the retailer's PL needs. Conversely, manufacturers on which the retailer is more dependent are likely to already have an influential relationship with the retailer (Ter Braak, Dekimpe, and Geyskens 2013) and the retailer is less likely to be able to push for PL supply. These effects are less likely to stretch to non-NB categories.

These observable characteristics and their expected associations with PL supply in NB categories are shown in the middle box of Figure 1. It is also plausible that some of these characteristics are endogenous and may be influenced by PL supply. We incorporate this possibility in our Study 1 model.

### Outcomes for the NBs of Manufacturers That Supply PL

As some of our discussion thus far has highlighted, supplying a PL to a retailer in a category should, in theory, help NB manufacturers improve the competitive position of their NBs through channel coordination and category management. NB manufacturers may also hope that supplying a PL will generate goodwill from the retailer in the form of more shelf space for its NBs.

However, do such benefits actually materialize for the NB manufacturers that supply a PL to a retailer (the box on the far right of Figure 1)? Kumar and Steenkamp (2007, p. 136) note that they “were never able to uncover hard evidence” to support such arguments. Ter Braak, Deleersnyder, et al. (2013) subsequently provide empirical evidence that a discount retailer that is beginning to add some NBs to its primarily PL assortment is more likely to stock a leading manufacturer's NBs if the manufacturer supplies its PL. As we note in the introduction, whether such distribution benefits from PL supply generalize across different types of retailers and manufacturers is an open question. It is also an open question whether PL supply affects the share of category sales that the dual brander's NBs get at the retailer.

#### Impact of PL supply on dual brander's NB distribution depth at the retailer

Most grocery retailers carry a large assortment of NBs, and expanding distribution depth of a PL supplier's NBs is likely to come at the expense of competing NBs on the shelf. Considerations of how much distribution depth the PL supplier's NBs already have and how strong demand has been from the retailer's clientele are therefore important. It is not clear how these considerations stack up against any potential goodwill. Also, if PL supply is a reciprocal action by manufacturers that are already well placed at the retailer, how much more of a boost can PL supply provide? Retailers may also have different PL sourcing strategies. If some source from many different dual branders and frequently change suppliers whereas others build enduring relationships with a few, any NB distribution benefits of PL supply may vary across retailers.

Finally, if the NBs of a dual brander that starts supplying PL to a retailer get a boost in distribution depth, then one has to also ask whether there is a distribution depth detriment when the PL supply arrangement is terminated. With limited shelf space in physical stores, if PL suppliers benefit from goodwill, those that stop supplying (and are presumably replaced by other suppliers) may be hurt by potential ill will. Thus, we leave as an empirical question what effect, if any, starting or ending PL supply to a retailer has on the distribution depth of dual brander NBs in the category.

#### Impact of PL supply on dual brander's NB share at the retailer

How much distribution depth different NBs have is directly controlled by the retailer. So, if the retailer chooses to reward the NBs of its PL supplier as a goodwill gesture, it can do so. However, what share of the retailer's category sales those NBs get is not directly under the control of the retailer or the manufacturer. Several factors drive sales, most importantly consumer preference. There is no reason to believe that consumer preference for a manufacturer's NBs will increase because it supplies PLs, or that it will decline if it stops supplying PLs. Greater influence in category management can help, but the objective of category management is to improve the performance of the whole category at the retailer and not to opportunistically advantage individual manufacturers (Gooner, Morgan, and Perreault 2011). Therefore, it is unclear whether supplying a PL to the retailer will increase the share of a dual brander's NBs and whether terminating a PL supply relationship will hurt the share of its NBs.

Whether the NB manufacturer supplies a PL to the retailer is only one factor affecting the NBs’ outcomes at the retailer. Some of the drivers of PL supply discussed previously also may have a direct association with those outcomes. Further, as discussed, while PL supply may be motivated by the quest for category influence and/or goodwill at the retailer, it may also be a reciprocal response by a manufacturer whose NBs are doing well at the retailer. We incorporate these effects in our Study 2 model.

## Data and Descriptive Statistics

### Sources of Data

Our empirical analysis covers the CPG categories sold in six of the largest grocery retailers in Spain: Mercadona, Dia, Carrefour, Eroski, Alcampo, and El Corte Inglés (including its Hipercor banner). We obtain data pertaining to the identity of the firms that supply PLs to each of these retailers in each CPG category for two years (2012 and 2017). These data come from Publicaciones Alimarket SA, a leading data provider in Spain. We also obtain household panel data for the 2008–2017 period covering all the CPG categories that GfK tracks, each of which has a unique EAN (European Article Number). The categories span four departments: food, beverages, personal care, and household products. Web Appendix A explains how we compile and merge data from the two sources. We have 272 categories in the data after dropping those in which most products are unbranded and those with no PL sales, and after combining some pairs of very similar GfK categories that are treated as one in the Alimarket data. The Alimarket data also define a “Family,” which can contain multiple related categories. For example, categories like cured ham and chorizo sausage are within the “prepared meat” family. We conduct our analyses in both Study 1 and Study 2 at the NB manufacturer-category-retailer level.

### Descriptives of the Six Retailers

The included retailers account for over 75% of total 2010 sales in our data.^1^ Their positioning in the market, our coverage of their data, and their PL suppliers are summarized in Table 1. The first panel shows that the retailers vary significantly in their market share, relative pricing, and PL shares. The second panel shows that our coverage of categories and PL suppliers is good. The number of categories in which PL suppliers are identified varies from 176 to 237 for the six retailers. In total, across the six retailers, we have 266 categories in which PL suppliers are identified for one or more retailers.

**Table 1.** Descriptives for Six Retailers.

**Variable**		**Retailer**					
		**Mercadona**	**Dia**	**Carrefour**	**Eroski**	**Alcampo**	**El Corte Inglés**
**Retailer Market Share and Pricing (Measured in 2010, Based on Purchases of Panelists)^a^**							
Volume market share	33.7%		15.2%	13.4%	7.3%	4.4%	1.8%
Price index^b^	69.8%		70.2%	82.3%	83.2%	80.5%	114.5%
NB price index^c^	95.3%		95.6%	100.0%	106.6%	90.3%	124.6%
PL price index^d^	63.9%		57.3%	59.2%	61.3%	56.9%	83.0%
PL volume share of sales	65.2%		66.8%	42.3%	52.3%	29.6%	25.0%
**Data Coverage (Measured in 2012)**							
No. of categories sold	272		269	272	271	272	271
No. of categories with PL	254		253	265	257	249	257
No. of categories with PL suppliers identified	220		199	211	237	176	188
**PL Suppliers (Measured in 2012)**							
Total no. of PL suppliers	76		228	326	249	169	225
Percentage of PL suppliers that are dual branders (DBs)	72.4%		77.2%	75.8%	83.1%	85.2%	86.2%
No. of NB categories in which DBs supply PL^e^	1.96 (1.83)		1.03 (.89)	1.15 (1.05)	1.25 (.91)	1.30 (1.02)	1.55 (1.48)
No. of non-NB categories in which DBs supply PL^e^	2.50 (3.72)		.61 (.99)	.54 (.84)	.59 (1.39)	.56 (.97)	.49 (.81)
No. of other retailers for which DBs supply a PL in same NB category^e^	.32 (.75)		1.19 (1.15)	1.08 (1.10)	.97 (1.12)	1.29 (1.15)	.77 (1.04)
No. of other retailers for which DBs supply a PL in same non-NB category^e^	.11 (.49)		.78 (.98)	.66 (.96)	.54 (.90)	.67 (.96)	.37 (.77)

^a^ Weighted averages across categories. SKU volume sales are used as weights to compute weighted average within a category. Category value sales are used as weights to compute weighted average across categories. These figures may differ from market shares reported in other sources due to differences in market definition, panel used, and other factors.

^b^ Retailer's price indexed to Carrefour's NB price.

^c^ Same as note b but for retailer's NB price only.

^d^ Same as note b but for retailer's PL price only.

^e^ Average with standard deviation in parentheses.

The third panel shows that well over 70% of PL suppliers are dual branders (versus suppliers that do not have NBs in the Spanish grocery market). It also shows that retailers differ in their PL sourcing strategies. In particular, Mercadona, which is the market leader and has a high PL share, relies on a much smaller number of PL suppliers. Compared with suppliers at other retailers, Mercadona's suppliers supply to it in more categories, and more of these are non-NB categories. They are also more likely to supply exclusively to Mercadona. On average they supply to only .32 other retailers in the same NB category that they supply to Mercadona, whereas the corresponding number for the five other retailers ranges from .77 to 1.29. It is interesting to note that Dia, which is also a PL-focused discount retailer, does not appear to follow a sourcing strategy similar to Mercadona's. Overall, this market provides a valuable opportunity to obtain generalizable insights regarding the supply side of PLs.

### Descriptives of Dual Branders

As will become clear when we specify the models for our two studies, we need to measure the drivers of PL supply in 2010–2013. Therefore, we include in our sample all 3,480 NB manufacturers that exist in the market during those years across the 266 categories. Table 2 provides descriptives of dual branders among these manufacturers and their PL supply patterns.

**Table 2.** Descriptives of PL Supply by Dual Branders.

**Variable**	**Overall**
Total no. of NB manufacturers	3,480
No. of NB categories per NB manufacturer^a^	2.09 (3.32)
No. of dual branders	554
No. of NB categories per dual brander^a^	3.70 (4.72)
No. of NB categories in which dual brander supplies PL^a^	1.70 (1.59)
No. of Non-NB categories in which dual brander supplies PL^a^	.71 (1.57)
Percentage of NB category cases in which dual branders supply PLs to two retailers	17.4%
Percentage of NB category cases in which dual branders supply PLs to more than two retailers	13.4%
Percentage of non-NB category cases in which dual branders supply PLs to two retailers	10.4%
Percentage of Non-NB category cases in which dual branders supply PLs to more than two retailers	4.8%

^a^ Means are listed with standard deviations in parentheses. 2012 PL supply data.

Even though most of the PL suppliers are NB manufacturers as shown in Table 1, Table 2 shows that dual branders account for only 15.9% (554 of 3,480) of NB manufacturers. Dual branders have NBs in more categories than average (3.70 vs. 2.09), and, on average, they supply PLs in 1.70 NB categories and .71 non-NB categories. Finally, most dual branders supply PLs in a category to only one of the retailers. The incidence of supply to two or more retailers is only 30.8% in NB categories and even lower (15.2%) in non-NB categories. This is consistent with the by-retailer figures we saw in Table 1.

To further examine PL supply in the dual branders’ NB and non-NB categories, we look at the 1,338 manufacturer-category observations in which the 554 dual branders supply PLs. The dual brander has NBs (1) in the same category in 942 or 70.4% of the cases; (2) in the same family in 20.1% of the cases (e.g., PL cured ham and NB chorizo sausage in the “prepared meat” family), (3) in the same department in 6.3% of the cases (e.g., PL kitchen foil and NB cleaning cloths in the “household products” department), and (4) not in the same department in 3.2% of the cases (e.g., PL olive oil and NB personal care products in different departments). Thus, PL supply outside NB categories is mostly in the same family as the NBs. This makes sense as manufacturers are less likely to have expertise in categories far removed from their NB experience.

Informed by this pattern, we define the consideration set for PL supply by a manufacturer in Study 1 as all its NB categories plus all categories within the same families as its NB categories, though we also check robustness with a broader definition.

## Study 1: Drivers of PL Supply by a NB Manufacturer to a Retailer in a Category

### Model Specification and Estimation

We next specify our model to estimate the association with PL supply of the manufacturer, retailer, and dyad characteristics identified in the “Conceptual Development” section. The unit of analysis is NB manufacturer m in category c within its consideration set at retailer r. The characteristics are measured in 2010, and PL supply is observed in 2012, as the superscripts in Equations 1 and 2 signify:

(1) $$\begin{array}{rl} {\mathrm{PLSup}}_{\mathrm{mcr}}^{12*}= & \beta_{0r}+\beta_{1r}{\mathrm{NoNB}}_{\mathrm{mc}}^{10}+\overset{8}{\underset{j=1}{\sum }}\;\beta_{\left(j+1\right)r}{\mathrm{ManufChar}}_{j}^{10}+\overset{3}{\underset{k=1}{\sum }}\;\beta_{\left(k+9\right)r}{\mathrm{RetChar}}_{k}^{10}\\ & +\overset{5}{\underset{l=1}{\sum }}\;\beta_{\left(l+12\right)r}{\mathrm{DyadChar}}_{l}^{10}+{\mathrm{NoNB}}_{\mathrm{mc}}^{10}\\ & *\left(\overset{8}{\underset{j=1}{\sum }}\;\beta_{\left(j+17\right)r}{\mathrm{ManufChar}}_{j}^{10}+\overset{3}{\underset{k=1}{\sum }}\;\beta_{\left(k+25\right)r}{\mathrm{RetChar}}_{k}^{10}+\overset{5}{\underset{l=1}{\sum }}\;\beta_{\left(l+28\right)r}{\mathrm{DyadChar}}_{l}^{10}\right)\\ & +\overset{3}{\underset{n=1}{\sum }}\;\beta_{\left(n+33\right)r}\mathrm{DeptF}E_{n}+\overset{5}{\underset{o=1}{\sum }}\;\beta_{\left(o+36\right)r}{\mathrm{Copula}}_{o}^{10}+\varepsilon_{\mathrm{mcr}}^{12}. \end{array}$$

(2) $${\mathrm{PLSup}}_{\mathrm{mcr}}^{12}=1\;\;\mathrm{if}\;\;{\mathrm{PLSup}}_{\mathrm{mcr}}^{12*}>0;\;0\;\mathrm{otherwise}.$$

In this probit model, ${\mathrm{PLSup}}_{\mathrm{mcr}}^{12*}$ is a latent variable, $\varepsilon_{\mathrm{mcr}}^{12}$ is normally distributed, and the specific manufacturer $\left({\mathrm{ManufChar}}_{j}^{10}\right)$ , retailer $\left({\mathrm{RetChar}}_{k}^{10}\right)$ , and dyad $\left({\mathrm{DyadChar}}_{l}^{10}\right)$ characteristics as well as department fixed effects (DeptFE_n_) are listed and defined in Table 3.^2^ The interactions with ${\mathrm{NoNB}}_{\mathrm{mc}}^{10}$ allow for different effects if m does not have a NB in c.

**Table 3.** Variable Definitions.

**Variable**		**Label**	**Definition^a^**
**Dependent Variable (Measured in 2012)**			
PL supply by manufacturer m to retailer r in category		PLSup_mcr_	1 if manufacturer m supplies PL in category c to retailer r in 2012, else 0
**Manufacturer Characteristics (Measured in 2010)**			
No NB of manufacturer m in category		NoNB_mc_	1 if manufacturer m does not have NB in category c but has NB in one or more other categories within the same family as c, else 0
NB growth			
Manufacturer m's growth		Growth_m_	Change in manufacturer m's volume share in category c from 2008 to 2010 divided by m's average volume share in c in 2008 and 2010; weighted average across all c in which m has NBs
Scale of NB production			
Manufacturer m's sales in category^b^		Sales_mc_	Value sales of manufacturer m in category c per 10,000 households; natural log
Manufacturer m's newness in category^b^		NewinCat_mc_	1 if manufacturer m has NBs in category c in 2010 but not in 2008, else 0
Breadth of NB production			
Manufacturer m's no. of NB categories		NumCats_m_	Number of distinct categories in which manufacturer m has NBs; natural log
Manufacturer m's no. of NBs per category		NBperCat_m_	Number of distinct NBs sold by manufacturer m across its categories divided by its number of categories
Differentiation of NBs			
Manufacturer m's price premium over PL in category^b^		PricePrem_mc_	Weighted average NB price per unit volume of manufacturer m over all retailers in category c divided by weighted average PL price per unit volume over all retailers in c; natural log
Manufacturer m's no. of new SKUs in category^b^		NewSKUs_mc_	Number of manufacturer m's new (since 2008) SKUs in category c; natural log
Premium and fighter NBs by manufacturer m in category^b^		Prem + Fighter_mc_	1 if manufacturer m has NBs in both high- and low-price tiers in category c, else 0 (high and low tiers are defined as top and bottom terciles respectively of the distribution of NB manufacturers’ price premiums over PL in c)
**Retailer Characteristics (Measured in 2010)**			
Size of retailer			
Retailer r's share of category sales		RShare_rc_	Volume sales of category c at retailer r divided by total volume sales of c in market
Size of PL business			
PL share of category at retailer r		PLShare_rc_	Volume sales of PL in category c at retailer r divided by total volume sales of c at r
Premium position of PL			
Retailer r's PL to NB price ratio in category		PLPriceRat_rc_	Weighted average PL price per unit volume at retailer r in category c divided by weighted average NB price per unit volume at r in c; natural log
**Dyad Characteristics (Measured in 2010)**			
Manufacturer's competition at retailer			
Competitive intensity for manufacturer m in category at retailer r^b^	CompIntensity_mcr_		Number of manufacturer m's competitors in category c stocked by retailer r divided by number of m's competitors in c
Manufacturer's NB prominence at retailer			
Distribution depth of manufacturer m in category at retailer r^b^		DistDepth_mcr_	Percentage of manufacturer m's SKUs in category c that are stocked by retailer r
Manufacturer m's share of retailer r's category sales^b^		Share_mcr_	Volume sales of manufacturer m's NBs in category c at retailer r divided by total volume sales of c at r
Manufacturer–retailer dependence			
Manufacturer m's dependence on retailer r		MDependonR_mr_	Share of manufacturer m's NB revenue over all of m's NB categories from retailer r
Retailer r's dependence on manufacturer m		RDependonM_mr_	Share of retailer r's revenue over all categories at r from manufacturer m's NBs
**Department Fixed Effects**			
Beverage category		Beverage_c_	1 if category c is in beverage department, 0 otherwise
Personal care category		PersonalCare_c_	1 if category c is in health/beauty/cosmetics department, 0 otherwise
Household products category		HHProd_c_	1 if category c is in household products department, 0 otherwise (reference department is food)

^a^ Wherever relevant, variables are computed based on sales in the whole market, not only the six retailers in our analysis. SKU volume sales are used as weights within a category, and value sales are weights across categories because volume sales are not comparable across categories.

^b^ When manufacturer M does not have NBs in category C, these are weighted averages of M's NBs in the same family as C, where weights are M's value sales in each category.

#### Endogeneity

As discussed previously, a PL can take the place of a fighter brand in the manufacturer's NB portfolio, and PL supply offers NB manufacturers and retailers the opportunity to coordinate the pricing of NBs and the PL in the category. Thus, having both a high-tier and a low-tier NB, the price premium of the manufacturer's NB, and the price ratio of the retailer's PL relative to NBs in the category may be outcomes in addition to being drivers of PL supply to a retailer. The same is true for the manufacturer's NB distribution depth and share at the retailer. These variables may drive PL supply to a retailer, be it in the hope of goodwill or in reciprocity to the retailer, but they may also be affected by PL supply. Thus, these five variables in the model for PL supply are likely to be endogenous based on the literature: $\mathrm{Prem}+{\mathrm{Fighter}}_{\mathrm{mc}}^{10}$ , ${\mathrm{PricePrem}}_{\mathrm{mc}}^{10}$ , ${\mathrm{PLPriceRat}}_{\mathrm{rc}}^{10}$ , ${\mathrm{DistDepth}}_{\mathrm{mcr}}^{10}$ , and ${\mathrm{Share}}_{\mathrm{mcr}}^{10}$ .

In the absence of suitable instruments, we include Gaussian copulas (Park and Gupta 2012) to control for their endogeneity. We check for nonnormality of each of these five variables since that is a key identifying assumption for the copula method (Papies, Ebbes, and Van Heerde 2017). The Shapiro–Wilk test rejects the null hypothesis of normality at *p* < .001 for all of them. Despite the inclusion of copulas and the temporal separation between measurement of the explanatory variables and observation of PL supply, we do not claim causal inference in Study 1 because the analysis is cross-sectional and PL supply could well have begun before 2012. The results from this study are best viewed as a test of theory-based associations.

#### Estimation

All continuous variables are mean-centered. We use the Gaussian copula control function approach to account for multiple endogenous variables (Park and Gupta 2012). All standard errors are clustered by both manufacturer and category. A pooling test rejects homogeneity of coefficient estimates across retailers. Since we have a large enough number of observations for each retailer, we estimate the probit model separately for each retailer instead of estimating retailer-specific parameters in one model. There are no multicollinearity concerns as the variance inflation factors are all well below 10. The highest variance inflation factor is only 2.05.

### Results

Overall summary statistics as well as sample sizes and parameter estimates for each retailer are provided in Web Appendix B. We report meta-analytic Zs (Rosenthal 1991; hereinafter metaZs) and weighted average marginal effects across retailers in Table 4 because our objective is to see if there is generalizable support of our theory-based expectations. All tests are two-tailed. Across the six retailers, the average pseudo R^2^ is .29.

**Table 4.** Drivers of PL Supply in NB and Non-NB Categories.

**Variable**	**Main Effect in NB Categories**		**Total Effect in Non-NB Categories^a^**	
	**Meta-Analytic Z**	**Weighted Average Marginal Effect^b^ (%)**	**Meta-Analytic Z**	**Weighted Average Marginal Effect^b^ (%)**
**Manufacturer Characteristics**				
NoNB_mc_	−13.77***	−4.16		
Growth_m_	−3.25***	−.48	−2.26***	−.02
Sales_mc_	7.99***	.98	3.39***	.05
NewinCat_mc_	−3.59***	−.42	.74	.03
NumCats_m_	.25	.02	5.61***	.07
NBperCat_m_	1.40	.12	2.77***	.03
PricePrem_mc_	−1.14	−.35	−.05	−.00
NewSKUs_mc_	5.23***	.52	−.92	−.01
Prem + Fighter_mc_	2.66***	.17	−.50	−.00
**Retailer Characteristics**				
RShare_rc_	−.25	.00	−1.34	−.01
PLShare_rc_	4.83***	.51	−.91	−.01
PLPriceRat_rc_	.30	.05	−.83	−.03
**Dyad Characteristics**				
CompIntensity_mcr_	3.46***	.27	3.04***	.02
DistDepth_mcr_	−4.36***	−.55	−2.76***	−.03
Share_mcr_	.69	.07	−.21	−.00
MDependonR_mr_	9.38***	.94	3.32***	.03
RDependonM_mr_	−3.48***	−.35	−1.43	−.04

*p < .1. **p < .05. ***p < .01.

^a^ Computed from Z-statistics of the variable's total effect (main coefficient plus interaction with NoNB_mc_).

^b^ Weights are inverse variances of marginal effects for each retailer. Marginal effect of a continuous variable is the change in probability of PL supply when other variables are at their means and the focal variable increases by one standard deviation from its mean. Marginal effect of a dummy variable is the corresponding change when the focal variable switches from 0 to 1.

We begin with several overarching findings. First, consistent with the descriptive patterns noted previously, NoNB_mc_ has a strongly negative association with PL supply. It has by far the highest metaZ (−13.77) and weighted average marginal effect (−4.16 percentage points). Second, within NB categories, the metaZs for most of the manufacturer and dyad characteristics are significant, whereas the retailer characteristics do not seem to matter, with the exception of PLShare_rc_. Third, the weighted average marginal effects of the drivers are small in absolute terms, but they are meaningful when compared with the baseline probability of PL supply, which is 3.74% in NB categories and .17% in non-NB categories. Fourth, as we expected, associations of most of the drivers with PL supply are weaker in non-NB than in NB categories. Notably though, many of them do matter even in non-NB categories.

#### Role of manufacturer characteristics

As expected, Growth_m_ is negatively associated with PL supply in NB categories (metaZ = −3.25). While weaker, the association remains significant even in non-NB categories (metaZ = −2.26). Manufacturers experiencing a slowdown in NB growth pursue PL revenue even in their non-NB categories.

Also as expected, scale is important for PL supply in NB categories. In fact, Sales_mc_ has the largest metaZ (7.99) and marginal effect (.98 percentage points) after NoNB_mc_. Manufacturers that are new to a category are also less likely to supply PL in it (metaZ for NewinCat_mc_ = −3.59). Sales_mc_ also matters for PL supply in non-NB categories (metaZ = 3.39), suggesting that perhaps large manufacturers can leverage their size and NB scale economies in related categories within the same family.

The breadth of NB production is not significantly associated with PL supply in NB categories (metaZ for NumCats_m_ and NBperCat_m_ = .25 and 1.40 respectively), but it is significant in non-NB categories (metaZs = 5.61 and 2.77 respectively). We did expect the association to be stronger in non-NB categories, but the difference is still notable. If the manufacturer has a NB in a category, it does not matter how many other categories it operates in. However, in supplying PL *beyond* its NB categories, breadth of categories matters more than anything else: NumCats_m_ has the largest metaZ and marginal effect of any driver in non-NB categories.

The results related to the role of NB manufacturer differentiation are interesting. Neither premium price, nor new SKU introductions, nor having both a premium brand and a fighter brand holds manufacturers back from PL supply. Although PricePrem_mc_ does not seem to matter, manufacturers that introduce more new SKUs of their NBs are *more* likely to supply PLs in their NB categories (metaZ for NewSKUs_mc_ = 5.23). This aligns more with the advantages of flexible production capabilities and retailers’ preference for innovative PL suppliers than with NB manufacturers guarding their innovations.^3^ Manufacturers also are *more* likely to supply PLs in their NB categories if they already have both premium and fighter NB offerings (metaZ for Prem + Fighter_mc_ = 2.66). Thus, manufacturers may not view a PL as a substitute for a low-priced fighter brand, as analytical models have theorized.

#### Role of retailer characteristics

Only one of the three retailer characteristics, PLShare_rc_, has a significant association with PL supply, and it is positive as expected (metaZ = 4.83). It is possible that the characteristics of a retailer may play a role in attracting certain types of NB manufacturers to supply PLs, even if they do not have a main effect. To explore this, we tested specific interactions between retailer and manufacturer characteristics that are plausible based on the literature. Complete model estimates are provided in Web Appendix B, but we highlight the major takeaways here.

First, there is a significantly positive interaction between Sales_mc_ and PLShare_mc_ in NB categories. Retailers whose PL business is large are *especially* likely to partner with large manufacturers in their NB categories (de Jong 2019). Second, the interaction of Prem + Fighter_mc_ with PLShare_rc_ is significantly positive, but its main effect becomes insignificant. Thus, the positive association with PL supply in NB categories that we documented for Prem + Fighter_mc_ occurs *only* when the retailer's PL share is large. Third, both the main effect of NewSKUs_mc_ and its interaction with PLPriceRat_rc_ are significantly positive. Thus, manufacturers that introduce new SKUs of their NBs are *even more* likely to also supply PLs in those categories when the retailer's PL is not heavily discounted and therefore has potential for reasonable margins. Finally, although the main effect of PricePrem_mc_ is not significant, its interaction with PLPriceRat_rc_ is significantly positive. These positive interactions with PLPriceRat_rc_ support the argument that differentiated manufacturers are more likely to supply PLs when there is potential for reasonable margin. They are also consistent with analytical predictions that it is optimal for differentiated NB manufacturers to supply PLs when the retailer has a premium PL offering (Gomez-Arias and Bello-Acebron 2008; Hara and Matsubayashi 2017), and with the empirical pattern that premium NBs are not hurt by introduction of premium PLs (Keller, Dekimpe, and Geyskens 2022).

#### Role of dyad characteristics

As expected, the intensity of competition from other NBs a manufacturer faces at a retailer is positively associated (metaZ for CompIntensity_mcr_ = 3.46) and the depth of its distribution at the retailer is negatively associated (metaZ for DistDepth_mcr_ = −4.36) with PL supply in NB categories. Also as expected, the more dependent a manufacturer is on the retailer for revenue, the more likely it is to supply PLs (metaZ for MDependonR_mcr_ = 9.38), and conversely, PL supply is less likely when the retailer is more dependent on the manufacturer (metaZ for RDependonM_mcr_ = −3.48). These results support the manufacturer's quest for category influence and goodwill. Many of these characteristics also play a role beyond the manufacturer's NB categories. The metaZs for CompIntensity_mcr_, DistDepth_mcr_, and MDependonR_mcr_ (3.04, −2.76, and 3.32 respectively) are significant in non-NB categories as well. It appears that manufacturers hope that supplying PLs in non-NB categories will have some spillover benefit for their NBs. Perhaps they also proactively pursue other revenue sources at the retailer as they see their NBs facing more intense competition and declining distribution depth.

Overall, our results from Study 1 offer support for some received wisdom regarding PL supply by NB manufacturers, reveal other associations that are contrary to it, and provide fodder for additional theoretical and empirical work. We elaborate on this in the “Discussion” section.

## Study 2: Association of PL Supply with NB Distribution and Share Outcomes

Among other things, Study 1 lends support to the idea that NB manufacturers are motivated to supply PLs with the hope that this would benefit their NBs at the retailer. The question, then, is whether this actually happens. Does entering into a PL supply arrangement with a retailer in a given category improve the distribution depth and share outcomes of a manufacturer's NBs in that category at that retailer? And conversely, does exiting a PL supply relationship have a deleterious effect? We next examine this issue by making use of the changes in PL supply status in our data between 2012 and 2017, the two years in which we observe PL supply. We first provide descriptives on the incidence of new PL supply arrangements (hereinafter “entries”) and terminated ones (hereinafter “exits”) that inform our modeling approach.

### Changes in PL Supply Over Time

Of the 3,480 NB manufacturers in our sample, the vast majority did not make a change in PL supply over the five years.^4^ We find that 2,818 (81.0%) did not supply PLs to any of the six retailers in either 2012 or 2017, and 127 (3.6%) continued to supply PLs with no change in the retailer-category combination(s) supplied. The remaining 535 NB manufacturers made a change, with 108 newly entering the PL business, 78 exiting it entirely, and 349 making changes in categories and/or retailers supplied while remaining in the PL business. We provide more detail on changes in PL supply at the manufacturer-category-retailer level in Table 5.

**Table 5.** Changes in Manufacturer-Category-Retailer Level PL Supply from 2012 to 2017.

**PL Supply Status in 2012**	**PL Supply Status in 2017**		**Total**
	**Don’t Supply PL**	**Supply PL**	
**In NB Categories**			
Don’t Supply PL	35,406	373	35,779
Supply PL	290	1,101	1,391
Total			37,170
**In Non-NB Categories in Same Families as NBs**			
Don’t Supply PL	173,490	168	173,658^a^
Supply PL	108	196	304
Total			173,962

^a^ The large N here derives from the categories in each NB manufacturer's additional consideration set for PL supply.

The top panel of Table 5 shows the total number of manufacturer-category-retailer level observations of the 3,480 NB manufacturers in their NB categories, and their PL supply status in both years. We observe no change in PL supply in over 98% of the cases (35,406 + 1,101 of 37,170). Among the 35,779 cases in which a NB manufacturer did not supply a PL to a given retailer in a given NB category in 2012, we see an entry by 2017 in 373 instances. And, among the 1,391 cases in which the NB manufacturer did supply a PL to a given retailer in a given NB category in 2012, we see an exit by 2017 in 290 instances. Thus, although the norm is no change in PL supply, we observe enough entries and exits in *total* to explore the consequences for the NB brands of the suppliers. The numbers of entries and exits at *individual* retailers, provided in Web Appendix C, are much smaller, with the fewest for Mercadona (15 entries and 18 exits).

The bottom panel of Table 5 shows the same information in categories where the manufacturer does not have NBs but that are in the consideration set for supplying PL. Here, the absolute number of entries (168 instances) and exits (108 instances) across the six retailers is substantially smaller. Therefore, we focus only on NB categories in our analysis of the effects of entries and exits on NB outcomes.

Table 6 elaborates further on the entries and exits within NB categories reported in Table 5. Among the 373 entries, only 28% (103) are by NB manufacturers that did not have any PL business with these retailers in 2012. Among the remaining 72%, some (98) are new entries in the focal category, whereas the rest (132 + 40) were previously supplying to other retailers in the category. Among the 290 exits, 35% (100) are by dual branders that entirely exit PL supply to these retailers, and another 23% (68) exit the focal category. The remaining 42% (78 + 44) of exits are by dual branders that remain in the PL business in the focal category through arrangements with other retailers. The last row of Table 6 shows that there are relatively few instances in which dual branders have an entry *as well as* an exit in the same category. Therefore, we examine the impact of entries and exits in separate models for simplicity.

**Table 6.** Types of PL Supply Entries and Exits in NB Categories.

**Decomposition of Entries**		**Decomposition of Exits**	
**Type of NB Manufacturers**	**No. of Entries**	**Type of NB Manufacturers**	**No. of Exits**
Entered PL supply overall	103	Exited PL supply overall	100
Entered PL supply in focal category but not overall	98	Exited PL supply in focal category but not overall	68
Added PL supply to retailer(s) without dropping others in focal category	132	Exited PL supply to retailer(s) without adding others in focal category	78
Added PL supply to retailer(s) and dropped others in focal category	40	Exited PL supply to retailer(s) and added others in focal category	44
Total	373	Total	290

### Model Specification and Estimation

Our goal is to model the impact of entry/exit by NB manufacturer m in NB category c at retailer r on the distribution depth and share outcomes of m's NBs in c at r. We use the 35,779 observations in the top row of Table 5 to model the impact of entry. We define indicator variable PLEntry^13∼17^_mcr_ = 1 for the 373 cases of entry and 0 otherwise, where the superscript 13∼17 signifies that the entry occurred sometime in the 2013–2017 period. Analogously, we use the 1,391 observations in the second row of Table 5 to model the impact of exit, defining PLExit^13∼17^_mcr_ = 1 for the 290 cases of exit and 0 otherwise.

In a nutshell, our strategy is to use a difference-in-difference approach to estimate the impact of entries/exits on changes in relative distribution and share outcomes of PL suppliers’ NBs from 2013 to 2017. To account for potential endogeneity of entries/exits, we specify probit models for them as functions of changes from 2010 to 2013 in the drivers of PL supply.^5^ The probit models provide control functions that can be included in the outcome models.

Details of the probit models for PLEntry^13∼17^_mcr_ and PLExit^13∼17^_mcr_ are provided in Web Appendix C. In addition to changes in the Study 1 manufacturer, retailer, and dyad characteristics from 2010 to 2013 (ΔManufChar^13–10^, ΔRetChar^13–10^, ΔDyadChar^13–10^), they include two other explanatory variables: the number of other retailers and other categories in which the manufacturer supplied PLs in 2012. These variables capture the fact that those that were already in the PL business are more likely to expand into new arrangements. Our primary interest, however, is in the NB outcome models, which we detail subsequently.

#### Difference-in-difference model for NB outcomes

We first compute the specific outcome variables of interest: the *relative* distribution depth and *relative* share of m's NBs at r in c, that is, m's distribution depth (DistDepth_mcr_) and share (Share_mcr_) in c at retailer r minus the corresponding average across the six retailers. We want to estimate the effect of entry/exit on changes in these relative outcomes from before to after entry. Thus, our dependent variables are the change in relative distribution depth (ΔRelDistDepth^17–13^_mcr_) and in relative share (ΔRelShare^17–13^_mcr_) from the first possible year when entry/exit could have occurred (i.e., 2013) to after (i.e., 2017). We model these differences in relative outcomes as a function of PLEntry^13∼17^_mcr_ and PLExit^13∼17^_mcr_ respectively, the corresponding generalized residuals discussed subsequently, and other relevant variables. The equation for the effect of PLEntry^13∼17^_mcr_ on ΔRelDistDepth^17–13^_mcr_ is next. Equations for the effect of entry on ΔRelShare^17–13^_mcr_ and of exit on ΔRelDistDepth^17–13^_mcr_ and ΔRelShare^17–13^_mcr_ are analogous.

(3) $$\begin{array}{rl} \;{\Delta \mathrm{RelDistDepth}}_{\mathrm{mcr}}^{17-13}= & \beta_{0r}+\beta_{1r}{\mathrm{PLEntry}}_{\mathrm{mcr}}^{13∼17}\\ & \;+\beta_{2r}{\mathrm{GenResidEntry}}_{\mathrm{mcr}}^{13∼17}\\ & \;+\overset{3}{\underset{k=1}{\sum }}\;\beta_{(k+2)r}\Delta {\mathrm{RetChar}}_{k}^{13-10}\\ & \;+\overset{5}{\underset{l=1}{\sum }}\;\beta_{(l+5)r}\Delta {\mathrm{DyadChar}}_{l}^{13-10}\\ & \;+\varepsilon_{\mathrm{mcr}}^{17-13}. \end{array}$$

#### Endogeneity

In Equation 3, GenResidEntry^13∼17^_mcr_ is the generalized residual from the probit model for PLEntry^13∼17^_mcr_. Since improving outcomes for NBs is one of the motivators for supplying PLs, entries and exits are likely to be endogenous. The generalized residual serves as a control function to account for that endogeneity (Wooldridge 2015).

It is plausible that changes in retailer (ΔRetChar^13–10^) and dyad (ΔDyadChar^13–10^) characteristics that affect entry/exit may also affect outcomes for the manufacturer's NBs, so we include those as explanatory variables. There is no reason to expect changes in the manufacturer characteristics, which are retailer-invariant, to directly affect changes in outcomes for the manufacturer's NBs at a given retailer r over and above the average across retailers, other than through the change in PL supply at r. The same is true for the two variables capturing previous PL supply to other retailers and in other categories that are included in the entry and exit models. Thus, these variables serve as reasonable exclusion restrictions in our outcome models.

In summary, we have temporal separation between changes in drivers and changes in PL supply (i.e., entries/exits) as well as between changes in PL supply and changes in outcomes; separation between the NB outcomes at the focal retailer versus the market; and a control function with defensible exclusion restrictions to account for the endogeneity of entries/exits.^6^

#### Estimation

Equation 3 is estimated by ordinary least squares with bootstrapped standard errors that are clustered by both manufacturer and category. As in Study 1, we test for pooling across retailers. Because there are so few instances of entries and exits for each retailer, it is more efficient to allow for retailer-specific parameters for the variables for which homogeneity across retailers is rejected than to estimate separate models for each retailer.

### Results

Table 7 summarizes the focal parameters of interest in the four outcome models (two outcomes each for entry and exit). These are β_1r_ and β_2r_ in Equation 3. The former estimates the impact of entry/exit, and the latter provides a test for the endogeneity of entry/exit. Pooling is not rejected for either of these parameters though it is rejected for some other parameters, which we therefore allow to be retailer-specific. Complete estimates are provided in Web Appendix D.

**Table 7.** NB Distribution Depth and Share Outcomes of PL Supply Entry and Exit.

**Variable**	**Effect on Δ Relative Distribution Depth**		**Effect on Δ Relative Share**	
	**Coefficient**	**t**	**Coefficient**	**t**
**Effect of Entering PL Supply to Retailer in a Category on Manufacturer's NBs**				
PLentry_mrc_	.12***	2.87	−.02	−.99
GenResidEntry_mrc_	−.04**	−2.43	.01	.97
**Effect of Exiting PL Supply to Retailer in a Category on Manufacturer's NBs**				
PLexit_mrc_	−.17**	−2.46	.00	.12
GenResidExit_mrc_	.09**	2.25	−.00	−.27

*p < .1. **p < .05. ***p < .01.

#### Impact on relative distribution depth

The first two columns in Table 7 show that entry is associated with a 12 percentage point increase (*p* < .01) in relative distribution depth for the manufacturer's NBs in the category at the retailer over the four years from 2013 to 2017. On the flip side, exit is associated with a 17 percentage point decrease (*p* < .05) in relative distribution depth for a manufacturer's NBs. The average distribution depth in 2012 is 16.9% for observations in the entry model and 25.9% for observations in the exit model. Thus, both the entry and the exit effects are sizeable though they occur over a period of four years. The signs and significances of the generalized residual tell us that these effects would have been significantly underestimated if we had not accounted for the endogeneity of entry and exit.

Although the small number of entries and exits makes it difficult to reliably estimate heterogeneity in their effects, we did explore moderation by the previous prominence of the NB manufacturer and the competitive intensity it faces at the retailer. The complete details of the model are provided in Web Appendix C, but we note here the two significant moderator effects. NB manufacturers whose distribution depth at the retailer was already increasing prior to entry (i.e., from 2010 to 2013) gain less from entering into a PL supply arrangement. In contrast, NB manufacturers benefit more from entering into a PL supply arrangement when the retailer was previously adding more NB competitors on its shelf. The magnitudes of both moderator effects are meaningful, but neither makes the positive effect on distribution depth insignificant within the range of the data.^7^ None of these moderators are significant for the effects of PL supply exit.

#### Impact on relative share

The second pair of columns in Table 7 shows that neither starting nor stopping PL supply has a significant effect on a NB manufacturer's relative share at a retailer. Recall that we expected any share benefit of supplying PLs to be weaker than for distribution depth because distribution is in the direct control of the retailer whereas sales are not.

Despite the insignificant average effect on relative share, we did check the same moderators, as shown in Web Appendix C. Only one, the previous change in the manufacturer's NB share at the retailer, is significant. However, the magnitude of the interaction is so small that the moderator would need to change by more than six standard deviations from its average to make the effect of either entry or exit on relative share significant.

In sum, Study 2 shows that, when a manufacturer starts supplying PLs to a retailer in its NB category, its NB distribution depth at that retailer relative to its average in the market gets a substantial boost. Conversely, when an existing PL supply arrangement is terminated, the NB manufacturer's relative distribution depth suffers. The boost from entry is higher for those that need it more. Those that were gaining distribution depth at the retailer prior to entry see a somewhat smaller boost while those facing increasing competition at the retailer see a slightly bigger boost. Equally importantly, there is no evidence that PL supply entry or exit has an effect on the NB manufacturer's relative share at the retailer.

## Discussion

Dual branding is a consequential practice the study of which has hitherto been limited by lack of data. To our knowledge, this is the first broad-based empirical analysis of the observable drivers of dual branding and of the effect of starting or terminating PL supply to a retailer on the distribution depth and share of the dual brander's NBs at that retailer. By jointly examining a comprehensive set of potential drivers, we are able to assess their relative roles. The findings we report are buttressed by several robustness checks detailed in Web Appendix D.

Our work helps researchers by revealing where theory is supported and where there is fodder for theoretical refinement and empirical testing. For managers, it offers a way to structure their own thinking about PL supply and help them build competitive intelligence into the PL decisions of their channel counterparts and competitors. As some of the managers with whom we shared our findings noted, they know their own PL supply decisions but our research helps them understand how different types of competitors and retailers may think about the PL business.

We focus on effects that generalize across different types of retailers, but it is worth noting that we examined differences between retailers and found that most effects are remarkably consistent. The model estimates for Study 1 by retailer are in Web Appendix B, Table WB3. It shows that the magnitudes and significance levels of coefficients do vary across retailers (hence the rejection of pooling). However, among all the parameters, there is only one instance in which a significant main effect has opposite signs for two retailers, and less than a handful of instances in which a significant interaction with NoNB_mc_ has opposite signs for two retailers. In Study 2, pooling across retailers is not rejected in either the entry or the exit model. In the NB outcome models, pooling of the parameters of interest, that is, effects of entry and exit, is not rejected although some other parameters differ across retailers.

We do find a different PL sourcing strategy for PL-focused retailer Mercadona. It uses a much smaller number of dual branders that supply PLs to it across several NB and non-NB categories, and its PL arrangements are even more long-lived than those of other retailers. In Study 1, the drivers of PL supply do not show *different* effects for Mercadona, but the statistical significance is weaker. Mercadona also contributes the least to Study 2 because of its small number of new PL supply entries and exits. It is worth noting that Mercadona's PL sourcing strategy does not seem to be emblematic of PL-focused retailers in general. The other PL-focused discount retailer, Dia, is more similar to other retailers than to Mercadona.

Should data permit, it would be fruitful for future research to delve into the PL arrangements of individual retailers across categories and suppliers, and empirically disentangle the preferences of the supplier and the retailer. However, there are likely many factors, unobservable to any but the two negotiating parties, that influence whether a PL arrangement exists between them. Thus, it would be a tall order to accurately predict which manufacturer supplies a PL to a particular retailer in a particular category, and especially which party terminates PL supply. Our goal was therefore not to predict, but to test the role of a comprehensive set of theory-based manufacturer, retailer, and dyad characteristics in PL supply and to assess its potential benefits for the NBs of dual branders in a generalizable way.

In the remainder of this concluding section, we highlight five thematic areas in which our findings are new or reveal nuances in how received wisdom aligns with empirical reality. Within each theme, we discuss some implications for research and practice.

### The Incidence and Longevity of PL Supply Arrangements in NB and Non-NB Categories

It is common knowledge that dual branding occurs, but our research reveals that (1) it is the rule, not the exception, with more than 70% of the PL suppliers in our data being NB manufacturers, and (2) dual branders supply PLs not just in their NB categories but also in categories where they do not have NBs. That the latter happens 30% of the time is especially worth underscoring for both researchers and managers. The substantial theoretical literature that offers rationales for dual branding is set in the context of categories in which the manufacturer has NBs and can get the benefits of category management and coordination for the NBs. Future theoretical development should also consider the decision of manufacturers to supply PLs in non-NB categories and the decision of retailers to source PLs from them. Some of the managers with whom we shared our findings were not previously aware that NB manufacturers supply PLs in non-NB categories as often as they do, whereas others told us their company supplies PLs in some non-NB categories because they see PLs as a growth engine.

We also document that dual branders generally supply PLs to only one or two retailers in a category, and most arrangements are long-lived. De Jong (2019) note anecdotally that these agreements can extend over a long term for PL-focused discounters, and our data on PL supply changes over a five-year period provide generalizable supporting evidence across a range of retailers.

For manufacturers, using PLs to opportunistically grow revenue and fill capacity while remaining focused on NBs is challenging (Kumar and Steenkamp 2007), even more so because most PL supply arrangements persist over time. In their NB categories, manufacturers should think about the role of PLs in their portfolio before signing contracts for PL supply to which they may be tethered for multiple years. Some manufacturers do this well. For example, a prominent organic food manufacturer we have shared our research with requires one to three years to convert new farms to organic production. It sells its output as nonorganic PLs during that period and also builds into its supply contracts the flexibility to walk away if the margins are not sufficient. Indeed, it did just that with a major U.S. warehouse club chain. Even for such manufacturers, our finding that termination of a PL supply arrangement has a detrimental effect on distribution depth for a dual brander's NBs is cautionary.

Our work suggests that supplying PLs in non-NB categories may be one way some dual branders try to strike a balance between revenue from PLs and focus on NBs. They limit this practice mostly to categories within the same families as their NBs, where they presumably have some market knowledge and/or production advantages. Anecdotally, we note several cases in our data of manufacturers producing PLs in non-NB categories that share major ingredients or similar processing knowhow with their NBs (e.g., facial tissue and paper towels, beauty cream and body milk, pasta and flour, frozen prepared meals and frozen prepared seafood). We also speculate that terminating PL supply arrangements may be somewhat easier in non-NB categories. The absolute number of entries and exits in non-NB categories in our data is too small to reliably assess potential spillover impact on NBs. However, on a percentage basis, 36% (108 of 304) of PL supply arrangements in non-NB categories are terminated within five years whereas the corresponding percentage in NB categories is 21%. Manufacturers may also view PLs as a way to test the waters or iron the wrinkles out of their production and logistics before launching NBs in new categories. Future research should study whether NB launches in new categories are more successful if they are preceded by PL supply and the extent to which they benefit from production experience, market insights, and easier access to distribution.

### The Role of Scale and Scope of NB Production Experience

The theoretical literature frequently cites production cost advantages as being important in PL supply but does not provide much insight beyond that. As far as scale is concerned, our research shows that the size of the manufacturer's NB business is one of the two most important drivers of PL supply in both NB and non-NB categories. Large manufacturers whose NB growth is slowing can exploit their scale to be efficient producers of PLs and NBs. We find that this is especially important for retailers that have high PL share and want the assurance that there is sufficient capacity to fulfill their demand (De Jong 2019). This does not mean most PL suppliers are large. After all, in most categories, there are many more small manufacturers than large ones, and they seek growth and influence with the retailers on which they are dependent. All else equal though, the probability that a large manufacturer will supply PLs is greater than the corresponding probability for a small manufacturer. Contrary to what some authors have written, PL supply is by no means only the purview of fringe manufacturers.

As far as scope of production is concerned, manufacturers with multiple NBs in multiple categories are more likely to supply PLs in non-NB categories. Indeed, multicategory NB experience is the single biggest driver of PL supply in non-NB categories among the 16 drivers that we examined. Within NB categories, we do not see a significant association with PL supply cross-sectionally, but as the number of categories in which the manufacturer has NBs increases, so does the likelihood of entering into new PL supply arrangements and continuing existing ones in NB categories. We are not aware of any prior empirical evidence on the role of portfolio breadth in the PL supply decision, nor has it been a factor in theoretical models that generally focus on NB and PL decisions within single categories. Thus, our findings are both novel and generalizable. They suggest that breadth of production should be considered in future theory development about PL supply, as should considerations of how production advantages can play out within and beyond the categories in which manufacturers have NBs.

For both manufacturers and retailers, it is important to understand what aspects of scale and scope can be leveraged in dual branding and stretched beyond NB categories. Scope economies can arise from a variety of production skills, but there is also the benefit of demand-side knowledge across a variety of related categories. And manufacturers may see potential economies in purchasing and processing of raw materials when the non-NB categories in which they supply PLs have ingredients in common with their NB categories. Indeed, prior research shows that purchasing costs as a percentage of sales are significantly lower for large players in a market (Ailawadi, Farris, and Parry 1999). While manufacturers that supply PL are likely aware of their competitive (dis)advantages, the retailers that do business with them and competing manufacturers that are considering dual branding would benefit from such insights.

### The Nuanced Relationship Between NB Differentiation and PL Supply

The headline from our analysis of the role of NB differentiation is that it does not hold manufacturers back from pursuing dual branding. However, the specific associations are nuanced and depend on the retailer's PL business. Manufacturers that introduce more new SKUs of their NBs are more likely to also supply PLs in the same category. The same is true for those that have both premium and fighter NB offerings, as long as the retailer's PL share is large. Both findings are contrary to received wisdom but make sense when viewed from the perspective of production flexibility. The first shows that there is capacity and flexibility to switch between different product variants, a requirement for PL supply. PLs may also be a way for the manufacturer to spread its fixed cost of product development over a larger sales base. The second suggests that the know-how to produce both premium and low-cost products is an advantage, and adding a substantial PL business to the mix can build scale. However, neither of these manufacturer characteristics is associated with greater PL supply in non-NB categories. Clearly, there is a difference between multicategory production experience (which is associated with greater PL supply in non-NB categories) and production flexibility within a category. This underscores the need for greater specificity in future theory development.

On average, manufacturers of premium-priced NBs are neither less nor more likely to supply PLs, but the odds that they will supply a PL are higher when the margin opportunity is reasonable. Manufacturers that lack experience in producing low-price NBs will have a harder time getting margins out of a PL when it is sold at a big discount. Retailers can try to persuade differentiated NB manufacturers to supply their PL by raising the price of the PL and creating margin opportunity for the supplier. In the process, they may also benefit themselves given recent findings that a smaller price gap with NBs is beneficial for long-term PL share (Gielens et al. 2023). Differentiated manufacturers can, for their part, also try to persuade retailers that emphasizing PLs too much is not as profitable for them as they might assume (e.g., Ailawadi and Harlam 2004; Ailawadi, Pauwels, and Steenkamp 2008). Indeed, a manager at a premium NB manufacturer that also supplies PL, but at prices comparable to its NB, told us they try to do just that. This also suggests a future opportunity to examine which types of dual branders supply different PL tiers (premium, standard, economic) in their NB categories and beyond.

### Goodwill, Reciprocity, and NB Distribution Benefits for Dual Branders

In combination, our results provide important insights on this issue that should be useful to researchers and managers alike. Overall, we find evidence supporting all three: (1) the quest for goodwill, (2) potential reciprocity, and (3) NB benefits. In support of the quest for goodwill, we find that manufacturers whose NBs have low distribution depth at a retailer and that are more dependent on the retailer for revenue are more likely to supply a PL. This is consistent with manufacturers’ motivation to influence retailer category management and build closer ties in the hope of benefiting their NBs. In support of potential reciprocity, we find that an increase in the distribution depth and share of a manufacturer's NBs at a retailer increases the probability that the manufacturer will subsequently start supplying a PL to the retailer in that category. And, in support of NB benefits, after controlling for endogeneity of the decision, we find that the NBs of manufacturers that start supplying a PL to a retailer enjoy a meaningful bump in relative distribution depth at that retailer. The bump is somewhat higher for manufacturers that were previously experiencing declining distribution depth and higher competitive intensity.

These findings are suggestive of the cycles of reciprocity discussed in the channels literature that can strengthen relationships over time (Lund, Kozlenkova, and Palmatier 2016). Equally importantly, we find that dual branders that exit PL arrangements suffer a meaningful detriment in the relative distribution depth of their NBs. Thus, terminating a PL arrangement could set off a cycle of negative reciprocity that can weaken relationships over time. Future research can model such cycles of harmful and beneficial reciprocity. Also, we explore heterogeneity in the effects of entries and exits on NB outcomes, but we are constrained by their small number. Future research that includes a more comprehensive analysis of how the effects vary across categories and manufacturers would be valuable. Given the longevity of PL supply arrangements, this would require a long time series of data.

### The Lack of NB Share Benefits for Dual Branders

Although we did not expect a strong NB share benefit, the fact that we find no share benefit despite a substantial distribution depth benefit warrants further thought. After all, shelf space elasticities are typically positive (Eisend 2014). We offer a few explanations. First, adding more SKUs of a brand does not have as big an effect on sales as does expanding distribution to more stores. At least some consumers who buy the newly stocked SKUs may simply be switching from preexisting SKUs of the brand in the store (Ailawadi and Farris 2020; Datta, Ailawadi, and Van Heerde 2017). Second, recall that previous increases in competitive intensity positively moderate the distribution depth benefit. When more competing NBs get on the shelf, it is harder for a manufacturer to gain share despite a boost to its own distribution depth. Third, NB manufacturers that were previously gaining share at the retailer are more likely to start supplying PLs (Web Appendix C, Table WC2), so there may simply be less room for a further boost in share. Still, there may be some situations in which dual branders enjoy a share benefit when they start supplying PLs or lose share when they stop supplying PLs. In one of our robustness tests (Web Appendix D, Table WD5), we do find a marginally significant reduction in share when a NB manufacturer exits PL supply. With data on more entries/exits over a longer period, future research can examine this and also model the dynamic relationship between distribution depth and share changes induced by PL supply.

The value of additional research notwithstanding, our finding does caution against viewing PL supply as a panacea for fading NBs. No retailer will continue adding SKUs of a NB that lacks sufficient consumer demand to justify the expansion. NB manufacturers should supply PLs if they can succeed in the PL business as an efficient and flexible producer at scale. Those with weak NBs may be better off migrating completely into PL production and perhaps merging with a dedicated PL business to benefit from economies of scale.

We conclude with the hope that this empirical analysis of dual branding spurs more theoretical and empirical research on the supply side of PLs.

## Supplemental Material

sj-pdf-1-jmx-10.1177_00222429231196575 - Supplemental material for Dual Branding by National Brand Manufacturers: Drivers and Outcomes Supplemental material, sj-pdf-1-jmx-10.1177_00222429231196575 for Dual Branding by National Brand Manufacturers: Drivers and Outcomes by Yu Ma, Kusum L. Ailawadi, Mercedes Martos-Partal and Óscar González-Benito in Journal of Marketing

## Associate Editor

Els Gijsbrechts

## Notes

1 The Lidl share in our data is 4.1%, but we exclude it because its PL suppliers are identified in only a few categories.

2 We found concave effects for several variables when we added quadratic terms, but none showed a sign reversal within the range of the data. Therefore, we use a natural log transformation, which reduces skewness and also parsimoniously captures nonlinearity.

3 We note that NewSKUs_mc_ counts the number of new SKUs irrespective of their innovativeness. Ter Braak et al. (2013), who find a negative association, count innovative new products, but recall that they only consider leading manufacturers at a discount retailer.

4 Recall that we examine their PL business in these six retailers only.

5 Recall that, in Study 1, drivers measured in 2010 explained PL status in 2012. Thus, changes in drivers from 2010 to after 2012 should explain changes in PL supply that occurred during 2013–2017.

6 We use change in drivers from 2010 to 2012 and change in outcomes from 2012 to 2017 in a robustness check (Web Appendix D).

7 A one standard deviation greater increase in prior distribution depth (competitive intensity) at the retailer reduces (increases) the benefit of entry by 3.4 (1.2) percentage points.
