# Supplementary material for: Dual Branding by National Brand Manufacturers: Drivers and Outcomes
Source: J Mark. 2023 Nov 15;88(3):69–87. doi: 10.1177/00222429231196575 (PMC13038171; doi:10.1177/00222429231196575)
Supplement: sj-pdf-1-jmx-10.1177_00222429231196575 - Supplemental material for Dual Branding by National Brand Manufacturers: Drivers and Outcomes [file sj-pdf-1-jmx-10.1177_00222429231196575.pdf]

Web Appendix to "Dual Branding by National Brand Manufacturers: Drivers and Outcomes"

Yu Ma (yu.ma@mcgill.ca), Kusum L. Ailawadi (kusum.ailawadi@dartmuth.edu), Mercedes Martos-Partal (mmartos@usal.es), Óscar González-Benito (oscargb@usal.es)

Table of Contents

Web Appendix A: Merging Data Across Two Sources page 2

Web Appendix B: Drivers of PL Supply to a Retailer by a NB Manufacturer page 4

- Table WB1: Summary Statistics page 4
- Table WB2: Correlation Matrix page 5
- Table WB3: Model Estimates by Retailer page 6
- Table WB4: Marginal Effects by Retailer page 9
- Table WB5: Meta-Analytic Results for Model with Interactions page 10
- Table WB6: Estimates by Retailer for Model with Interactions page 11

Web Appendix C: Effect of PL Supply Entries and Exits on NB Outcomes page 15

- Table WC1: Number of Entries & Exits in NB Categories by Retailer page 15
- Probit Specification for Entry and Exit Models page 15
- Table WC2: Estimates of Entry and Exit Probit Models page 17
- Table WC3: Estimates of NB Outcome Models page 18
- Table WC4: Estimates of NB Outcome Models with Interactions page 22

Web Appendix D: Robustness Checks page 26

- Summary page 26
- Table WD1: Multivariate Probit Model Estimates for PL Supply page 28
- Table WD2: Meta-Analytic Zs for Indep. vs. MVP PL Supply Model page 30
- Table WD3: Meta-Analytic Zs with 2011 vs. 2010 PL Supply Drivers page 31
- Table WD4: Entry and Exit Models with 2010-2012 Changes in Drivers page 32
- Table WD5: 2012-2017 Distribution Depth and Share Outcome Models page 33

These materials have been supplied by the authors to aid in the understanding of their paper. The AMA is sharing these materials at the request of the authors.

Web Appendix A: Merging Data Across Two Sources

Private Label Supplier Data

Publicaciones Alimarket, SA. provided a downloadable database called MDD ALIMARKET RESEARCH containing the identities of PL suppliers for various grocery retailers in Spain. The database includes (a) the name of each PL supplier, (b) the categories in which it supplies PL identified at up to three levels of specificity – "family", "product category", and, in some cases a narrower "product variety", (c) the name(s) of retailers to whom it supplies PL in each category, and (d) the PL name. For example, 3M Spain supplies the Hipercor brand to El Corte Inglés in the cleaning accessories family in four product categories (cleaning cloths, scouring pads, mops, and gloves).[1]

Scanner Data

Home-scan purchase data from GfK include a retailer name and ID, category name and ID and manufacturer name and ID. We matched each retailer name in the GfK data to retailer names in the Alimarket data and inserted the GfK retailer ID in the latter. Similarly, we matched each GfK category name to Alimarket and inserted the GfK Category ID in the Alimarket database. In some cases, the GfK category matched the Alimarket "product category", in others it matched the Alimarket "family", and in some it matched the Alimarket "product variety". For example, the GfK beer category matched with Alimarket's beer "family", olives matched with Alimarket's olives "product category", and drain cleaner matched with Alimarket's drain cleaner "variety". In a few instances, we combined GfK categories because they were very similar and Alimarket didn't differentiate between them (for example, liquid and solid air freshener). Finally,

[1] Alimarket also provides reports about each firm in its database, from which we determine whether the firm sells NBs in the Spanish grocery retail market, only in other markets, or not at all. When we merge the Alimarket data with GFK data, the accuracy of this coding is double-checked as only the first set is present in the GfK data.

we matched the list of GfK manufacturer names with the list of Alimarket PL supplier names (also checking their parent firm/corporate group names when necessary) and inserted the corresponding GfK manufacturer IDs in the Alimarket database. After this, we could merge the two databases by retailer, category, and manufacturer ID.

Web Appendix B: Drivers of PL Supply to a Retailer by a NB Manufacturer

Table WB1: Summary Statistics

	Variable	Mean	SD
PL Supply by Manufacturer M to Retailer R in Cat	$PLSup_{mrc}$	.01	.09
No NBs of Manufacturer in Cat	$NoNB_{mc}$	.78	.42
NB Growth	$Growth_{m}$	.27	.81
Scale of NB Production	$Sales_{mc}$	4.56	2.00
	$NewinCat_{mc}$	.11	.32
Breadth of NB Production	$NumCats_{m}$	.72	.81
	$NBperCat_{m}$	1.14	.99
Differentiation of NBs	$PricePrem_{mc}$	.58	.70
	$NewSKUs_{mc}$	.96	.89
	$Prem{+}Fighter_{mc}$	.08	.27
Size of Retailer	$RShare_{rc}$	.12	.12
Size of PL Business	$PLShare_{rc}$	.52	.27
Premium Position of PL	$PLPriceRat_{rc}$	-.55	.45
Manufacturer's Competition at Retailer	$CompIntensity_{mcr}$	.23	.10
Manufacturer's NB Prominence at Retailer	$DistDepth_{mcr}$	.19	.30
	$Share_{mcr}$	.02	.06
Manufacturer-Retailer Dependence	$MDependonR_{mr}$	.08	.19
	$RDependonM_{mr}$	.00	.00

Note: Means and standard deviations are calculated at the appropriate unit of analysis for each variable, e.g., total number of unique NB manufacturers for *M* level variables (N = 3,480), total number of unique manufacturer-category combinations for *M-C* level variables (N = 7,237), total number of unique manufacturer-category-retailer combinations for *M-C-R* level variables (N = 37,170).

Table WB2: Correlation Matrix

		V1	V2	V3	V4	V5	V6	V7	V8	V9	V10	V11	V12	V13	V14	V15	V16	V17
$NoNB_{mc}$	V1	1.00																
$Growth_{m}$	V2	.05	1.00															
$Sales_{mc}$	V3	.08	-.12	1.00														
$NewinCat_{mc}$	V4	-.02	.60	-.29	1.00													
$NumCats_{m}$	V5	-.24	-.10	.17	-.08	1.00												
$NBperCat_{m}$	V6	.04	-.05	.22	-.09	-.24	1.00											
$PricePrem_{mc}$	V7	-.02	-.01	-.05	.04	.09	-.08	1.00										
$NewSKUs_{mc}$	V8	-.01	.13	.48	.05	.20	.22	-.04	1.00									
$Prem+Fighter_{mc}$	V9	-.03	-.03	.22	-.08	.11	.31	-.07	.27	1.00								
$RShare_{rc}$	V10	.01	.00	.00	.00	.01	.00	.01	.00	.00	1.00							
$PLShare_{rc}$	V11	.03	.00	-.02	-.01	-.01	-.01	-.03	.00	.02	.40	1.00						
$PLPriceRat_{rc}$	V12	.00	.04	.01	.02	-.05	-.02	-.11	.00	.02	-.01	-.22	1.00					
$CompIntensity_{mcr}$	V13	.06	-.01	.11	.01	-.06	.04	.02	-.01	-.02	-.07	-.19	-.08	1.00				
$DistDepth_{mcr}$	V14	.06	-.04	.30	-.05	.03	-.02	.06	-.09	-.03	-.03	-.06	-.02	.21	1.00			
$Share_{mcr}$	V15	-.02	-.07	.46	-.10	.20	.10	-.04	.28	.09	-.01	-.04	-.02	.01	.31	1.00		
$MDependonR_{mr}$	V16	.02	.03	.02	.03	-.04	.01	.02	-.01	-.01	.00	-.04	.02	.04	.24	.07	1.00	
$RDependonM_{mr}$	V17	-.09	-.07	.27	-.05	.37	.09	.04	.18	.06	-.04	-.04	-.03	.06	.21	.42	.05	1.00

Note: This is based on N = 211,132 (the complete set of observations used in our PL supply model, pooled across the six retailers).

Table WB3: Model Estimates by Retailer

**DV: $PLSup_{mcr}$**	**Mercadona**	**Dia**	**Carrefour**	**Eroski**	**Alcampo**	**El Corte Ingles**
$NoNB_{mc}$	-1.064*** (-4.50)	-1.224*** (-7.04)	-1.503*** (-10.78)	-1.650*** (-6.97)	-2.429*** (-4.30)	-.118 (-.14)
$Growth_{m}$	-.308* (-1.65)	-.092 (-1.59)	-.069 (-1.13)	-.105* (-1.86)	-.124 (-1.26)	-.037 (-.48)
$Sales_{mc}$	.097* (1.72)	.109*** (5.08)	.067*** (2.83)	.116*** (4.66)	.046** (2.00)	.086*** (3.30)
$NewinCat_{mc}$	-.245 (-1.07)	-.147 (-.90)	-.171 (-1.56)	-.297** (-2.53)	-.177 (-.97)	-.250* (-1.75)
$NumCats_{m}$	.066 (.83)	.001 (.01)	-.044 (-.89)	-.013 (-.25)	-.026 (-.47)	.101 (1.38)
$NBperCat_{m}$	-.021 (-.26)	.007 (.14)	.031 (.78)	.043 (.99)	.056 (1.06)	.044 (.71)
$PricePrem_{mc}$	.285 (.54)	-.466 (-1.10)	.094 (.51)	-.390 (-.95)	-.130 (-.24)	-.684 (-1.56)
$NewSKUs_{mc}$	-.065 (-.75)	.168*** (3.69)	.157*** (3.29)	.169*** (3.22)	.085 (1.43)	.103* (1.92)
$Prem+Fighter_{mc}$	-.297 (-1.48)	.369*** (2.74)	.012 (.09)	.200 (1.51)	.235 (1.60)	.271** (2.06)
$RShare_{rc}$	-.983 (-1.52)	3.029*** (2.99)	-.843 (-.81)	.455 (.36)	2.172 (.79)	-13.096** (-2.44)
$PLShare_{rc}$	.452 (1.60)	-.073 (-.33)	.426** (2.03)	.327* (1.73)	.930*** (3.96)	.461*** (2.84)
$PLPriceRat_{rc}$	.043 (.19)	-.286 (-.77)	-.069 (-.16)	.578 (1.06)	.397 (.75)	-.110 (-.34)
$CompIntensity_{mcr}$	.805 (1.07)	.412 (1.01)	.045 (.11)	.484 (1.18)	1.389*** (2.68)	1.030** (2.42)
$DistDepth_{mcr}$	-.348 (-.78)	-.678 (-1.60)	-.728** (-2.46)	-.786*** (-2.79)	-.366 (-1.02)	-.625** (-2.04)

$Share_{mcr}$	1.420**	-.226	.266	-.587	.373	-.242
	(2.26)	(-.23)	(.51)	(-.82)	(.56)	(-.58)
$MDependonR_{mr}$	2.046***	.932***	.150	1.511***	.707**	1.180***
	(6.40)	(3.35)	(.56)	(5.96)	(2.54)	(4.16)
$RDependonM_{mr}$	-33.918	-33.785	-20.990*	-32.309**	-15.917	-19.132
	(-1.06)	(-1.57)	(-1.80)	(-1.99)	(-.96)	(-1.15)
$Growth_{m}$ X $NoNB_{mc}$	-.042	-.091	-.064	.087	-.103	.146
	(-.26)	(-.69)	(-.61)	(.89)	(-.54)	(1.44)
$Sales_{mc}$ X $NoNB_{mc}$	.062	-.012	.018	.020	.019	-.134***
	(.77)	(-.20)	(.41)	(.40)	(.26)	(-2.80)
$NewinCat_{mc}$ X $NoNB_{mc}$	.778	.353	.473**	.249	.060	-.562
	(1.58)	(.34)	(2.53)	(.44)	(.07)	(-.42)
$NumCats_{m}$ X $NoNB_{mc}$	.228***	.235***	.141**	.189***	.123	.160
	(2.98)	(2.95)	(2.42)	(3.29)	(1.39)	(1.58)
$NBperCat_{m}$ X $NoNB_{mc}$	.056	.155**	.012	.091	-.031	.113
	(.74)	(1.97)	(.15)	(1.19)	(-.22)	(1.32)
$PricePrem_{mc}$ X $NoNB_{mc}$	.107	.213*	.107	.276***	.197	.123
	(1.00)	(1.77)	(1.43)	(2.95)	(1.44)	(.97)
$NewSKUs_{mc}$ X $NoNB_{mc}$	.164	-.244***	-.201**	-.272***	-.287**	.061
	(1.25)	(-2.73)	(-2.33)	(-3.18)	(-2.25)	(.63)
$Prem+Fighter_{mc}$ X $NoNB_{mc}$	-.619	-.694**	.021	-.256	.256	-.263
	(-1.12)	(-2.03)	(.11)	(-.89)	(.76)	(-.95)
$RShare_{rc}$ X $NoNB_{mc}$	.201	-2.400	2.356	-5.359*	-9.818	13.388*
	(.25)	(-1.29)	(1.56)	(-1.96)	(-1.56)	(1.70)
$PLShare_{rc}$ X $NoNB_{mc}$	-.533	.297	-.559*	-.600**	-1.132**	-.483
	(-1.60)	(.90)	(-1.68)	(-1.98)	(-2.57)	(-1.37)
$PLPriceRat_{rc}$ X $NoNB_{mc}$	-.118	.103	-.199	-.199	-.058	-.503***
	(-.82)	(.70)	(-1.19)	(-1.04)	(-.23)	(-3.31)
$CompIntensity_{mcr}$ X $NoNB_{mc}$	-.519	1.161	1.327	.479	2.495**	-2.006*
	(-.37)	(1.35)	(1.60)	(.43)	(2.45)	(-1.94)

$DistDepth_{mcr}$ X $NoNB_{mc}$	.560	-.055	-.393	-1.168	-3.646**	.804
	(.72)	(-.09)	(-.59)	(-1.28)	(-2.41)	(1.63)
$Share_{mcr}$ X $NoNB_{mc}$	-4.137	.768	-.450	.261	1.236	.496
	(-1.45)	(.63)	(-.41)	(.19)	(.19)	(.22)
$MDependonR_{mr}$ X $NoNB_{mc}$	-.552*	-.292	-.882	-.910***	-.680	-.779
	(-1.77)	(-.51)	(-1.31)	(-3.01)	(-1.43)	(-1.62)
$RDependonM_{mr}$ X $NoNB_{mc}$	-30.530	-.395	6.700	10.295	44.315	-175.196
	(-.77)	(-.01)	(.04)	(.22)	(.71)	(-1.11)
$Beverage_{c}$	.094	.068	-.126	-.300**	-.259	-.306
	(.34)	(.52)	(-.99)	(-2.40)	(-1.05)	(-1.55)
$PersonalCare_{c}$	-.369	-.406	-.527*	-.345	-.195	-.694***
	(-1.40)	(-1.50)	(-1.91)	(-1.36)	(-.88)	(-2.73)
$HouseholdProd_{c}$	.425*	.290**	.112	.291*	.295**	.014
	(1.83)	(2.11)	(.87)	(1.82)	(2.21)	(.10)
Copula $PricePrem_{mc}$	-.251	.006	-.092	.103	-.099	.309
	(-.81)	(.02)	(-.73)	(.42)	(-.32)	(1.16)
Copula $Prem+Fighter_{mc}$	.087*	-.027	.031	-.068**	-.018	-.090***
	(1.70)	(-.81)	(1.00)	(-2.29)	(-.47)	(-2.79)
Copula $PLPriceRat_{rc}$	-.053	.022	.072	-.211	-.082	.109
	(-.52)	(.14)	(.48)	(-1.06)	(-.48)	(.80)
Copula $DistDepth_{mcr}$	-.079	.055	.027	.022	-.047	.072
	(-.90)	(.71)	(.41)	(.35)	(-.60)	(.97)
Copula $Share_{mcr}$	.113	.048	.195***	.126**	.202***	.195***
	(1.17)	(.95)	(3.39)	(2.29)	(2.84)	(3.63)
Constant	-2.380***	-2.122***	-1.627***	-1.765***	-1.704***	-3.023***
	(-12.66)	(-28.89)	(-23.02)	(-17.88)	(-7.02)	(-5.51)
Pseudo R^2	.32	.27	.27	.28	.28	.32
N	36,120	34,082	36,735	39,318	31,805	33,072

Note: t statistics in parentheses.
*** $p < .01$; ** $p < .05$; * $p < .10$; two-tailed test.

Table WB4: Marginal Effects by Retailer

	Mercadona (%)	Dia (%)	Carrefour (%)	Eroski (%)	Alcampo (%)	El Corte Ingles(%)
In NB Categories						
$Growth_{m}$	-.75	-.40	-.43	-.56	-.60	-.23
$Sales_{mc}$	.57	1.13	1.00	1.46	.53	1.25
$NewinCat_{mc}$	-.25	-.27	-.45	-.66	-.36	-.64
$NumCats_{m}$	.21	.00	-.35	-.09	-.17	.79
$NBperCat_{m}$	-.05	.03	.19	.22	.26	.26
$PricePrem_{mc}$	.62	-1.70	.48	-1.75	-.53	-3.49
$NewSKUs_{mc}$	-.16	.72	.95	.87	.40	.61
$Prem+Fighter_{mc}$	-.19	.44	.02	.28	.30	.44
$RShare_{rc}$	-.32	.76	-.23	.07	.19	-.85
$PLShare_{rc}$	.40	-.08	.71	.47	.99	.69
$PLPriceRat_{rc}$	.07	-.60	-.22	1.37	.81	-.35
$CompIntensity_{mcr}$	.18	.18	.03	.23	.59	.58
$DistDepth_{mcr}$	-.17	-.60	-1.11	-.92	-.43	-.87
$Share_{mcr}$	.17	-.05	.08	-.15	.09	-.08
$MDependonR_{mr}$	1.27	.61	.23	1.66	.81	1.47
$RDependonM_{mr}$	-.17	-.52	-.62	-.69	-.32	-.52
In Non-NB Categories						
$Growth_{m}$	-.15	-.06	-.05	-.01	-.06	.03
$Sales_{mc}$	.17	.08	.08	.11	.04	-.03
$NewinCat_{mc}$	.10	.03	.05	-.01	-.01	-.09
$NumCats_{m}$	.17	.10	.05	.08	.03	.09
$NBperCat_{m}$	.01	.05	.02	.05	.01	.04
$PricePrem_{mc}$	.15	-.07	.07	-.03	.01	-.13
$NewSKUs_{mc}$	.04	-.02	-.02	-.04	-.05	.04
$Prem+Fighter_{mc}$	-.11	-.03	.00	-.01	.03	.00
$RShare_{rc}$	-.05	.01	.03	-.05	-.04	.00
$PLShare_{rc}$	-.01	.02	-.01	-.03	-.01	.00
$PLPriceRat_{rc}$	-.02	-.03	-.05	.06	.04	-.09
$CompIntensity_{mcr}$	.01	.05	.05	.03	.09	-.02
$DistDepth_{mcr}$	.02	-.05	-.11	-.15	-.25	.01
$Share_{mcr}$	-.06	.01	.00	-.01	.02	.00
$MDependonR_{mr}$	.16	.03	-.07	.04	.00	.02
$RDependonM_{mr}$	-.06	-.04	-.03	-.03	.03	-.24

Table WB5: Meta-Analytic Results for Model with Interactions

**Variable**	**Main Effect in NB Cats.**		**Total Effect in Non-NB Cats.** [a]	
	Meta Analytic Z	Wtd. Avg. Marginal Effect[b] (%)	Meta Analytic Z	Wtd Avg. Marginal Effect[b] (%)
Manufacturer Characteristics				
$NoNB_{mc}$	-13.27***	-3.52		
$Growth_{m}$	-3.15***	-.46	-2.30**	-.02
$Sales_{mc}$	7.17***	.96	2.97***	.05
$NewinCat_{mc}$	-3.72***	-.44	.70	.03
$NumCats_{m}$	.26	.02	5.64***	.07
$NBperCat_{m}$	1.67*	.14	2.76***	.03
$PricePrem_{mc}$	-.78	-.13	.39	.01
$NewSKUs_{mc}$	5.74***	.56	-1.03	-.02
$Prem{+}Fighter_{mc}$	1.60	.18	.38	.00
Retailer Characteristics				
$RShare_{rc}$	-.21	.01	-1.37	-.01
$PLShare_{rc}$	3.21***	.65	.82	.01
$PLPriceRat_{rc}$	.51	.21	-.34	-.02
Dyad Characteristics				
$CompIntensity_{mcr}$	3.35***	.26	2.91***	.02
$DistDepth_{mcr}$	-3.75***	-.43	-2.38**	-.03
$Share_{mcr}$	1.71*	.11	-.25	-.00
$MDependonR_{mr}$	9.28***	.93	3.36***	.03
$RDependonM_{mr}$	-3.35***	-.34	-1.52	-.04
Manufacturer-Retailer Interactions[c]				
$PLShare_{rc}$ x $Sales_{mc}$	4.29***	.42	-4.34***	-.03
$PLShare_{rc}$ x $Prem{+}Fighter_{mc}$	1.93*	.19	1.21	.01
$PLPriceRat_{rc}$ x $PricePrem_{mc}$	5.53***	.34	2.47**	.02
$PLPriceRat_{rc}$ x $NewSKUs_{mc}$	3.93***	.23	-.89	-.01

[a] Computed from the Z-statistics of variable's total effect (main coefficient plus interaction with $NoNB_{mc}$).
[b] Weights are inverse variances of marginal effects for each retailer. Marginal effect of a continuous variable is the change in probability of PL supply when other variables are at their means and the focal variable increases by one std. dev. from its mean. Marginal effect of a dummy variable is the corresponding change when the focal variable switches from 0 to 1.
[c] Marginal effect of an interaction is the change in the marginal effect of the Manufacturer variable when the Retailer variable increases by one std. deviation from its mean.

Table WB6: Estimates by Retailer for Model with Interactions

**DV: $PLSup_{mcr}$**	**Mercadona**	**Dia**	**Carrefour**	**Eroski**	**Alcampo**	**El Corte Ingles**
$NoNB_{mc}$	-1.178*** (-4.02)	-1.224*** (-6.72)	-1.504*** (-10.66)	-1.706*** (-6.84)	-2.403*** (-4.27)	.013 (.02)
$Growth_{m}$	-.325* (-1.69)	-.091 (-1.52)	-.067 (-1.11)	-.102* (-1.72)	-.126 (-1.28)	-.029 (-.38)
$Sales_{mc}$	.082 (1.36)	.102*** (3.48)	.070*** (2.85)	.096*** (3.81)	.079*** (2.73)	.105*** (3.32)
$NewinCat_{mc}$	-.279 (-1.12)	-.135 (-.84)	-.172 (-1.57)	-.333*** (-2.88)	-.177 (-.97)	-.247* (-1.73)
$NumCats_{m}$	.070 (.87)	.003 (.06)	-.044 (-.89)	-.014 (-.26)	-.027 (-.48)	.096 (1.35)
$NBperCat_{m}$	-.011 (-.13)	.011 (.25)	.037 (.90)	.053 (1.22)	.059 (1.13)	.044 (.71)
$PricePrem_{mc}$	.236 (.43)	-.431 (-1.13)	.181 (1.04)	-.188 (-.52)	-.074 (-.14)	-.700 (-1.61)
$NewSKUs_{mc}$	-.074 (-.81)	.196*** (4.22)	.175*** (3.54)	.201*** (3.70)	.091 (1.55)	.105* (1.87)
$Prem+Fighter_{mc}$	.073 (.06)	.109 (.69)	.051 (.42)	.042 (.32)	.323* (1.73)	.111 (.70)
$RShare_{rc}$	-1.011 (-1.53)	2.828*** (2.76)	-.778 (-.74)	1.063 (.75)	2.129 (.80)	-13.547** (-2.53)
$PLShare_{rc}$	.363 (1.15)	-.181 (-.80)	.300 (1.28)	.179 (.88)	.777*** (3.25)	.413** (2.10)
$PLPriceRat_{rc}$	.088 (.30)	.100 (.31)	-.242 (-.63)	.444 (.80)	.605 (.99)	-.182 (-.52)
$CompIntensity_{mcr}$	.776 (1.01)	.392 (.96)	-.025 (-.06)	.505 (1.17)	1.397*** (2.70)	1.086** (2.44)
$DistDepth_{mcr}$	.063 (.14)	-.557 (-1.58)	-.670*** (-2.63)	-.607* (-1.75)	-.350 (-.97)	-.678** (-2.41)

$Share_{mcr}$	1.377** (2.34)	.151 (.16)	.573 (1.07)	.065 (.08)	.627 (.89)	-.160 (-.35)
$MDependonR_{mr}$	2.047*** (6.28)	.958*** (3.38)	.126 (.47)	1.475*** (5.75)	.719*** (2.59)	1.171*** (4.25)
$RDependonM_{mr}$	-32.987 (-1.00)	-32.153 (-1.56)	-19.980* (-1.74)	-30.575** (-1.97)	-13.951 (-.84)	-18.148 (-1.09)
$PLShare_{rc}$ x $Sales_{mc}$	.077 (1.08)	.059 (.72)	.111 (1.44)	.335*** (4.13)	.196* (1.69)	.127 (1.44)
$PLShare_{rc}$ x $Prem+Fighter_{mc}$	.706 (.27)	.904 (1.64)	.485 (.95)	.372 (.71)	.727 (1.01)	.077 (.16)
$PLPriceRat_{rc}$ x $PricePrem_{mc}$	.110 (1.04)	.205 (1.30)	.250** (2.25)	.394** (2.51)	.444** (2.01)	.525*** (4.43)
$PLPriceRat_{rc}$ x $NewSKUs_{mc}$	.175 (1.53)	.261*** (3.06)	.175 (1.55)	.274*** (3.04)	.030 (.26)	.016 (.19)
$Growth_{m}$ X $NoNB_{mc}$	-.024 (-.15)	-.091 (-.70)	-.069 (-.66)	.083 (.84)	-.110 (-.58)	.137 (1.33)
$Sales_{mc}$ X $NoNB_{mc}$	.117 (1.30)	.015 (.22)	.016 (.36)	.049 (1.01)	-.068 (-.87)	-.204*** (-3.79)
$NewinCat_{mc}$ X $NoNB_{mc}$	.810 (1.63)	.336 (.33)	.476** (2.58)	.279 (.50)	.064 (.08)	-.597 (-.45)
$NumCats_{m}$ X $NoNB_{mc}$	.226*** (2.80)	.232*** (2.92)	.141** (2.43)	.189*** (3.30)	.132 (1.48)	.165 (1.63)
$NBperCat_{m}$ X $NoNB_{mc}$	.040 (.52)	.151* (1.91)	.008 (.10)	.084 (1.10)	-.037 (-.26)	.121 (1.40)
$PricePrem_{mc}$ X $NoNB_{mc}$	.166 (1.38)	.212* (1.74)	.093 (1.19)	.239*** (2.99)	.184 (1.32)	.179 (1.38)
$NewSKUs_{mc}$ X $NoNB_{mc}$	.169 (1.25)	-.285*** (-3.03)	-.229*** (-2.67)	-.299*** (-3.37)	-.294** (-2.22)	.039 (.40)
$Prem+Fighter_{mc}$ X $NoNB_{mc}$	-.413 (-.07)	-.712 (-.37)	.019 (.08)	-.288 (-.59)	.326 (.82)	-.117 (-.35)

$RShare_{rc}$ X $NoNB_{mc}$	.110<br>(.14)	-2.285<br>(-1.25)	2.351<br>(1.53)	-5.802**<br>(-2.03)	-9.588<br>(-1.56)	14.797*<br>(1.83)
$PLShare_{rc}$ X $NoNB_{mc}$	.195<br>(.40)	.445<br>(1.26)	-.338<br>(-.91)	-.170<br>(-.50)	-.974**<br>(-2.11)	-.317<br>(-.74)
$PLPriceRat_{rc}$ X $NoNB_{mc}$	-.099<br>(-.64)	.164<br>(.96)	-.119<br>(-.67)	-.179<br>(-.91)	-.195<br>(-.69)	-.410***<br>(-2.68)
$CompIntensity_{mcr}$ X $NoNB_{mc}$	-.394<br>(-.28)	1.108<br>(1.26)	1.352<br>(1.62)	.509<br>(.46)	2.514**<br>(2.35)	-2.142**<br>(-2.05)
$DistDepth_{mcr}$ X $NoNB_{mc}$	.637<br>(.80)	-.088<br>(-.14)	-.440<br>(-.66)	-1.147<br>(-1.21)	-3.705**<br>(-2.39)	.823<br>(1.58)
$Share_{mcr}$ X $NoNB_{mc}$	-4.452<br>(-1.40)	.463<br>(.38)	-.717<br>(-.63)	-.574<br>(-.42)	.999<br>(.15)	.250<br>(.10)
$MDependonR_{mr}$ X $NoNB_{mc}$	-.548*<br>(-1.68)	-.303<br>(-.53)	-.842<br>(-1.24)	-.866***<br>(-2.84)	-.684<br>(-1.45)	-.789*<br>(-1.68)
$RDependonM_{mr}$ X $NoNB_{mc}$	-36.589<br>(-.87)	-2.824<br>(-.06)	4.636<br>(.03)	8.275<br>(.18)	40.921<br>(.68)	-192.686<br>(-1.25)
$PLShare_{rc}$ X $Sales_{mc}$ X $NoNB_{mc}$	-.291**<br>(-2.49)	-.124<br>(-.88)	-.210*<br>(-1.65)	-.623***<br>(-4.91)	-.516**<br>(-2.52)	-.413**<br>(-2.25)
$PLShare_{rc}$ X $Prem+Fighter_{mc}$ X $NoNB_{mc}$	-1.382<br>(-.11)	-.008<br>(-.00)	-.389<br>(-.36)	.716<br>(.47)	.387<br>(.32)	.928<br>(.80)
$PLPriceRat_{rc}$ X $PricePrem_{mc}$ X $NoNB_{mc}$	.075<br>(.47)	.129<br>(.44)	.039<br>(.16)	-.081<br>(-.49)	-.520*<br>(-1.82)	-.512**<br>(-2.47)
$PLPriceRat_{rc}$ X $NewSKUs_{mc}$ X $NoNB_{mc}$	-.155<br>(-1.23)	-.313**<br>(-2.21)	-.265<br>(-1.54)	-.183<br>(-1.19)	-.119<br>(-.51)	-.216<br>(-1.40)
$Beverage_{c}$	.075<br>(.27)	.056<br>(.43)	-.128<br>(-1.03)	-.295**<br>(-2.36)	-.251<br>(-1.03)	-.312<br>(-1.64)
$PersonalCare_{c}$	-.342<br>(-1.28)	-.284<br>(-1.20)	-.435*<br>(-1.69)	-.193<br>(-.85)	-.146<br>(-.73)	-.777***<br>(-2.87)
$HouseholdProd_{c}$	.405*<br>(1.70)	.280**<br>(2.06)	.095<br>(.73)	.280*<br>(1.72)	.264*<br>(1.92)	.014<br>(.10)

Copula $PricePrem_{mc}$	-.226	.018	-.128	.032	-.135	.292
	(-.70)	(.08)	(-1.06)	(.15)	(-.46)	(1.12)
Copula $Prem+Fighter_{m}$	$-.123^{***}$	.002	.017	-.017	-.015	-.021
	(-2.91)	(.05)	(.63)	(-.55)	(-.39)	(-.72)
Copula $PLPriceRat_{rc}$	-.085	-.120	.118	-.163	-.111	.136
	(-.64)	(-.91)	(.88)	(-.82)	(-.60)	(.95)
Copula $DistDepth_{mcr}$	$-.194^{**}$	.042	.019	-.033	-.052	.066
	(-2.36)	(.57)	(.35)	(-.46)	(-.65)	(.79)
Copula $Share_{mcr}$	$.174^{**}$	.029	$.193^{***}$	$.154^{***}$	$.201^{***}$	$.228^{***}$
	(2.27)	(.51)	(3.22)	(2.65)	(2.79)	(3.65)
Constant	-2.417^{***}	-2.083^{***}	-1.629^{***}	-1.737^{***}	-1.737^{***}	-3.042^{***}
	(-12.18)	(-27.57)	(-22.60)	(-16.31)	(-7.34)	(-5.55)
Pseudo R^2	.33	.28	.27	.30	.29	.31
N	36,120	34,082	36,735	39,318	31,805	33,072

Note: t statistics in parentheses.
*** $p < .01$; ** $p < .05$; * $p < .10$; two-tailed test.

Web Appendix C: Effect of PL Supply Entries and Exits on NB Outcomes

Table WC1: Number of Entries and Exits in NB Categories by Retailer

	Sample for Entry Model		Sample for Exit Model	
**Retailer**	No PL Supply in either 2012 or 2017	PL Supply in 2017 but not 2012 (Entry)	PL Supply in both 2012 and 2017	PL Supply in 2012 but not 2017 (Exit)
Mercadona	6,124	15	98	18
Dia	5,819	104	147	53
Carrefour	5,994	69	239	69
Eroski	6,458	86	230	45
Alcampo	5,411	46	161	35
El Corte Inglés	5,600	53	226	70
Total	35,406	373	1,101	290

Probit Specification for Entry and Exit Models

Analogous to the PL supply model in Equation 1 of the main paper, we use a probit specification for entry. The specification for exit is identical with $PLExit_{mcr}$ as the dependent variable.

$$\begin{aligned} &\mathrm{PLEntry}_{mcr}^{13\sim17*} \\ &= \beta_{0r} + \sum_{j=1}^{7} \beta_{jr}\Delta\mathrm{ManufChar}_{j}^{13-10} + \sum_{k=1}^{3} \beta_{(k+7)r}\Delta\mathrm{RetChar}_{k}^{13-10} + \sum_{l=1}^{5} \beta_{(l+10)r}\Delta\mathrm{DyadChar}_{l}^{13-10} \\ &+ \beta_{16}\mathrm{PLSupORetsSameCat}_{mc}^{13-10} + \beta_{17r}\mathrm{PLSupOCats}_{mc}^{13-10} \\ &+ \varepsilon_{mcr}^{13\sim17} \qquad \text{(WCE1)} \end{aligned}$$

$$PLEntry^{13\sim17}_{mcr} = 1 \text{ if } PLEntry^{13\sim17}_{mcr}{}^{*} > 0\text{; 0 otherwise} \qquad \text{(WCE2)}$$

where $PLEntry^{13\sim17}_{mcr}$ equals 1 if m started supplying PL to r in c sometime during 2013-2017, 0 otherwise, $PLEntry^{13\sim17}_{mcr}{}^{*}$ is a latent variable, and $\varepsilon^{13\sim17}_{mcr}$ is normally distributed. The manufacturer, retailer, and dyad characteristics are prefixed by Δ and have the superscript $^{13\text{-}10}$ to depict changes from 2010 to 2013 because we are modeling changes in PL supply made during the 2013-2017 period.

Compared with the PL supply model, note the absence of (a) $NoNB_{mc}$ and its interactions because this analysis is within NB categories; (b) copula terms because, unlike in the main model, we

are modeling changes over time and also have clear temporal priorness with changes in the drivers occurring before changes in PL supply; and (c) department fixed effects because the change analysis obviates their need. There are two additional explanatory variables – number of other retailers to which *m* supplied PL in c in 2012 ($PLSupORetsSameCat_{mc}$) and number of other categories in which m supplied PL in 2012 ($PLSupOCats_{mc}$) -- to account for the fact that NB manufacturers who were already in other PL supply arrangements are more likely to enter into new ones.

Table WC2: Estimates of Entry and Exit Probit Models

**Variable**	**PL Supply Entry Model**		**PL Supply Exit Model**	
	Coefficient	t-stat	Coefficient	t-stat
Δ $Growth_{m}$	.00	(.02)	.01	(.08)
Δ $Sales_{mc}$	$.05^{**}$	(2.20)	$-.10^{**}$	(-2.23)
Δ $NumCat_{sm}$	$.28^{***}$	(2.65)	$-.71^{***}$	(-3.76)
Δ $NBperCat_{m}$	.06	(.81)	.07	(.79)
Δ $PricePrem_{mc}$	.07	(1.23)	.15	(1.16)
Δ $NewSKUs_{mc}$	$.08^{**}$	(2.13)	-.06	(-.96)
Δ $Prem+Fighter_{mc}$	.07	(.76)	-.09	(-.92)
Δ $RShare_{rc}$	.91	(.76)	-1.60	(-.57)
Δ $PLShare_{rc}$	.02	(.10)	-.41	(-.80)
Δ $PLPriceRat_{rc}$	.04	(.49)	.00	(.00)
Δ $CompIntensity_{mcr}$	.62	(1.32)	-2.82^{***}	(-3.01)
Δ $DistDepth_{mcr}$	$.17^{**}$	(2.02)	-.27	(-1.26)
Δ $Share_{mcr}$	2.97^{***}	(4.54)	-.39	(-.47)
Δ $MDependonR_{mr}$	-.13	(-.50)	.60	(1.55)
Δ $RDependonM_{mr}$	6.60	(.28)	-64.03^{*}	(-1.65)
$PLSupORetsSameCat_{mc}$	$.78^{***}$	(10.81)	-.09	(-.96)
$PLSupOCats_{mc}$	$.23^{***}$	(4.93)	-.09	(-1.39)
Mercadona	$-.57^{***}$	-4.58	-.07	-.22
Dia	$.31^{***}$	3.59	$.28^{*}$	1.82
Carrefour	$.17^{**}$	2.06	.14	.96
Eroski	$.18^{**}$	2.16	-.06	-.42
El Corte Ingles	.05	.54	.19	1.26
Constant	-2.57^{***}	-35.44	$-.96^{***}$	-8.41
Pseudo R^2	.15		.07	
N	35,779		1,391	

*** p < .01; ** p < .05; * p < .10; two-tailed test.

Note: A pooling test did not reject homogeneity of coefficients across retailers, so we report estimates from the pooled model. Generally, we expect the same signs for various drivers as in the PL supply model for the entry model and the opposite signs for the exit model. Although several coefficients are not significant, likely due to limited longitudinal variation in the variables over the three-year period, the signs of the ones that are significant are largely in line with expectations.

Table WC3: Estimates of NB Outcome Models

Variable	Impact of Entry on		Impact of Exit on	
	Δ Rel. Dist. Depth	Δ Rel. Share	Δ Rel. Dist. Depth	Δ Rel. Share
$PLentry_{mrc}$	.118***	-.022		
	(2.87)	(-.99)		
$GenResidEntry_{mrc}$	-.044**	.008		
	(-2.43)	(.97)		
$PLexit_{mrc}$			-.172**	.002
			(-2.46)	(.12)
$GenResidExit_{mrc}$			.094**	-.003
			(2.25)	(-.27)
Δ $RShare_{rc}$	.062	-.002	-.327	-.231*
	(1.16)	(-.29)	(-.81)	(-1.75)
Δ $PLShare_{rc}$	.010	.006	.060	.005
	(.77)	(1.38)	(.91)	(.15)
Δ $PLPriceRat_{rc}$	-.007	.001	-.039	.012
	(-.34)	(.72)	(-1.26)	(1.33)
Δ $CompIntensity_{mcr}$	.126	.001	-.043	-.139**
	(1.37)	(.15)	(-.43)	(-2.23)
Δ $DistDepth_{mcr}$	-.325***	.001*	-.341***	.003
	(-36.06)	(1.81)	(-8.57)	(.44)
Δ $Share_{mcr}$	.225***	-.168***	.268*	-.232***
	(4.09)	(-3.51)	(1.73)	(-3.87)
Δ $MDependonR_{mr}$	.011	-.001	.126	.015
	(.20)	(-.42)	(1.55)	(.90)
Δ $RDependonM_{mr}$	-.073	.291	-18.776	1.455
	(-.03)	(.57)	(-.74)	(.58)
Mercadona	.039***	.001	.015	-.008
	(6.92)	(1.17)	(.50)	(-.56)
Dia	.043***	.003***	.039**	.003
	(7.50)	(3.79)	(2.00)	(.71)

Carrefour	.045*** (7.07)	.002*** (2.66)	.076*** (2.87)	.007 (1.00)
Eroski	.010 (1.53)	.003*** (4.79)	.041* (1.78)	.008 (1.24)
El Corte Ingles	-.003 (-.53)	.000 (.83)	-.007 (-.32)	.006 (1.10)
Mercadona x Δ $PLShare_{rc}$		-.008 (-1.51)		.190 (1.29)
Dia x Δ $PLShare_{rc}$		-.000 (-.01)		-.017 (-.38)
Carrefour x Δ $PLShare_{rc}$		-.008 (-1.42)		.032 (.75)
Eroski x Δ $PLShare_{rc}$		.007 (1.11)		.030 (.54)
El Corte Ingles x Δ $PLShare_{rc}$		-.001 (-.21)		.020 (.43)
Mercadona x Δ $PLPriceRat_{rc}$	.003 (.16)			
Dia x Δ $PLPriceRat_{rc}$	.007 (.32)			
Carrefour x Δ $PLPriceRat_{rc}$	.039 (1.52)			
Eroski x Δ $PLPriceRat_{rc}$	.036 (1.28)			
El Corte Ingles x Δ $PLPriceRat_{rc}$	-.002 (-.11)			
Mercadona x Δ $CompIntensity_{mcr}$	-.081 (-.80)			.303 (1.46)
Dia x Δ $CompIntensity_{mcr}$	-.241** (-2.29)			.184** (2.54)

Carrefour x Δ CompIntensity$_{mcr}$	-.216**<br>(-2.09)			.140<br>(1.41)
Eroski x Δ CompIntensity$_{mcr}$	-.159<br>(-1.44)			.140<br>(1.52)
El Corte Ingles x Δ CompIntensity$_{mcr}$	-.097<br>(-.80)			.235**<br>(2.39)
Mercadona x Δ Share$_{mcr}$		-.070<br>(-.57)		
Dia x Δ Share$_{mcr}$		.134*<br>(1.68)		
Carrefour x Δ Share$_{mcr}$		-.140*<br>(-1.91)		
Eroski x Δ Share$_{mcr}$		-.005<br>(-.07)		
El Corte Ingles x Δ Share$_{mcr}$		-.054<br>(-.48)		
Mercadona x Δ MDependonR$_{mr}$	.084<br>(1.36)			
Dia x Δ MDependonR$_{mr}$	.017<br>(.21)			
Carrefour x Δ MDependonR$_{mr}$	.154**<br>(2.31)			
Eroski x Δ MDependonR$_{mr}$	.019<br>(.22)			
El Corte Ingles x Δ MDependonR$_{mr}$	-.037<br>(-.43)			
Mercadona x Δ RDependonM$_{mr}$			17.835<br>(.47)	
Dia x Δ RDependonM$_{mr}$			-10.650<br>(-.44)	
Carrefour x Δ RDependonM$_{mr}$			-10.293	

			(-.29)	
Eroski x Δ $RDependonM_{mr}$			5.183<br>(.20)	
El Corte Ingles x Δ $RDependonM_{mr}$			24.870<br>(.98)	
Constant	-.032*** (-7.04)	-.000<br>(-.23)	.007<br>(.35)	-.004<br>(-.69)
Adjusted R^2	.12	.05	.11	.18
N	35,779	35,779	1,391	1,391

Note: In each model, retailer-specific coefficients are specified only for those variables where pooling is rejected.
t statistics in parentheses. *** p < .01; ** p < .05; * p < .10; two-tailed test.

Table WC4: Estimates of NB Outcome Models with Interactions

Variable	Impact of Entry on<br>Δ Rel. Dist. Depth	 Δ Rel. Market Share	Impact of Exit on<br>Δ Rel. Dist. Depth	 Δ Rel. Market Share
$PLentry_{mrc}$	.130*** (2.83)	.002 (.15)		
$PLentry_{mrc}$ X Δ $DistDepth_{mcr}$	-.130** (-2.38)	.010 (1.04)		
$PLentry_{mrc}$ X Δ $Share_{mcr}$	.018 (.09)	-.286** (-2.18)		
$PLentry_{mrc}$ X Δ $CompIntensity_{mcr}$	.243* (1.83)	-.004 (-.10)		
$GenResidEntry_{mrc}$	-.048** (-2.43)	-.001 (-.14)		
$PLexit_{mrc}$			-.162** (-2.23)	-.003 (-.22)
$PLexit_{mrc}$ X Δ $DistDepth_{mcr}$			-.004 (-.04)	-.007 (-.66)
$PLexit_{mrc}$ X Δ $Share_{mcr}$			.269 (.88)	.281* (1.89)
$PLexit_{mrc}$ X Δ $CompIntensity_{mcr}$			-.115 (-.43)	-.047 (-.75)
$GenResidExit_{mrc}$			.089** (2.10)	.001 (.10)
Δ $RShare_{rc}$	.062 (1.16)	-.00 (-.10)	-.338 (-.82)	-.215* (-1.78)
Δ $PLShare_{rc}$	.010 (.77)	.005 (1.31)	.059 (.95)	.025 (.73)
Δ $PLPriceRat_{rc}$	-.007 (-.36)	.001 (1.04)	-.036 (-1.15)	.013 (1.41)
Δ $CompIntensity_{mcr}$	.123	.000	-.028	.025

	(1.34)	(-.05)	(-.23)	(.78)
Δ $DistDepth_{mcr}$	-.324***	.001	-.341***	.002
	(-35.92)	(1.55)	(-7.04)	(.20)
Δ $Share_{mcr}$	.226***	-.161***	.223*	-.267***
	(4.00)	(-3.32)	(1.33)	(-3.80)
Δ $MDependonR_{mr}$	.011	-.001	.109	.009
	(.21)	(-.66)	(1.21)	(.65)
Δ $RDependonM_{mr}$	-.088	.247	-.556	1.308
	(-.04)	(.48)	(.05)	(.52)
Mercadona	.039***	.001	.013	-.006
	(6.91)	(1.55)	(.41)	(-.50)
Dia	.042***	.002***	.042**	.005
	(7.45)	(3.68)	(2.17)	(.96)
Carrefour	.045***	.002***	.071**	.008
	(7.05)	(2.63)	(2.44)	(1.19)
Eroski	.010	.003***	.039	.009
	(1.52)	(4.80)	(1.60)	(1.42)
El Corte Ingles	-.003	.000	-.008	.006
	(-.52)	(.82)	(-.38)	(1.19)
Mercadona x Δ $PLShare_{rc}$		-.007		.164
		(-1.44)		(1.09)
Dia x Δ $PLShare_{rc}$		-.001		-.034
		(-.12)		(-.76)
Carrefour x Δ $PLShare_{rc}$		-.008		.009
		(-1.42)		(.21)
Eroski x Δ $PLShare_{rc}$		.007		.015
		(1.12)		(.27)
El Corte Ingles x Δ $PLShare_{rc}$		-.001		-.000
		(-.11)		(-.01)
Mercadona x Δ $PLPriceRat_{rc}$	.004			
	(.18)			

Dia x Δ PLPriceRat$_{rc}$	.008	
	(.35)	
Carrefour x Δ PLPriceRat$_{rc}$	.040	
	(1.55)	
Eroski x Δ PLPriceRat$_{rc}$	.036	
	(1.29)	
El Corte Ingles x Δ PLPriceRat$_{rc}$	-.002	
	(-.09)	
Mercadona x Δ CompIntensity$_{mcr}$	-.079	
	(-.78)	
Dia x Δ CompIntensity$_{mcr}$	-.244**	
	(-2.33)	
Carrefour x Δ CompIntensity$_{mcr}$	-.215**	
	(-2.07)	
Eroski x Δ CompIntensity$_{mcr}$	-.160	
	(-1.45)	
El Corte Ingles x Δ CompIntensity$_{mcr}$	-.099	
	(-.81)	
Mercadona x Δ Share$_{mcr}$		-.021
		(-.21)
Dia x Δ Share$_{mcr}$		.154*
		(1.89)
Carrefour x Δ Share$_{mcr}$		-.126*
		(-1.74)
Eroski x Δ Share$_{mcr}$		-.004
		(-.06)
El Corte Ingles x Δ Share$_{mcr}$		-.062
		(-.56)
Mercadona x Δ MDependonR$_{mr}$	.084	
	(1.36)	

Dia x Δ $MDependonR_{mr}$	.017			
	(.21)			
Carrefour x Δ $MDependonR_{mr}$	.153**			
	(2.29)			
Eroski x Δ $MDependonR_{mr}$	.019			
	(.22)			
El Corte Ingles x Δ $MDependonR_{mr}$	-.038			
	(-.44)			
Constant	-.032***	-.000	.009	-.004
	(-7.03)	(-.67)	(.38)	(-.67)
Adjusted R^2	.12	.05	.11	.19
N	35,779	35,779	1,391	1,391

Note: In each model, retailer-specific coefficients are specified only for those variables where pooling is rejected.
t statistics in parentheses. *** $p < .01$; ** $p < .05$; * $p < .10$; two-tailed test.

Web Appendix D: Robustness Checks

Summary

We conducted several checks to ensure the robustness of our results. We list these below:

1. Variables in Model Specification
   a. The growth rate of a manufacturer can be measured at the manufacturer or the manufacturer-category level. We report results using the former in the paper but also estimated our PL supply models using the latter and found no substantive changes.
   b. We defined the consideration set for PL supply by a manufacturer as all its NB categories plus all categories within the same families as its NB categories. Expanding the consideration set to all categories within the same department as the manufacturer's NB categories exponentially increases the sample size with a very small incidence of PL supply in the expanded observations. However, we estimated our model with this expanded consideration set and our results remain substantively unchanged.
   c. We included interactions between changes in the relevant manufacturer and retailer characteristics (i.e., the interactions tested in the PL supply model) in the entry and exit models and found that they were mostly not significant and the other estimates remained substantively unchanged.
   d. In the entry and exit models, we used indicator variables for whether or not a NB manufacturer previously supplied PL to other retailers in the category and in other categories instead of the actual number in which it supplied. We found very similar results.
2. Alternative Model and Time Periods
   a. *Multivariate Probit (MVP) Specification for Drivers of PL Supply*: A MVP model would allow for error dependencies between the six retailers. However, it requires the same sample for each retailer. Since there are differences across retailers in the categories sold and the

categories for which we have PL supply information, we would have to drop all observations that are not common to all six retailers. This issue is exacerbated with the consideration set of categories for each NB manufacturer. Therefore, we report results on the basis of six by-retailer probit models in the paper. We did, however, estimate a MVP model using the common set of observations within NB categories and found a very similar pattern of results. The MVP estimates are provided in Table WD1. For ease of comparison, we show in Table WD2, the meta-analytic Zs from probit models as well as the same calculation from the MVP (we do recognize that meta-analytic Zs are intended to be used across independent models – this is simply for ease of comparison).

b. *Different Time Period for Measurement of Drivers of PL Supply*: We also estimated our PL Supply model using 2011 data on drivers instead of 2010. The results were very similar. The meta-analytic Zs for the NB category (main) and non-NB category (main plus interaction) effects are provided in Table WD3.

c. *Different Time Period for Entry, Exit, and Outcome Models*: Using the change in drivers from 2010 to 2012 in the entry/exit model and the change in outcomes from 2012 to 2017 creates cleaner temporal separation for the outcome models because 2012 is unambiguously before the entry/exit whereas it is possible that some entries/exits occurred in 2013. However, the trade-off is that it reduces the window for longitudinal variation in drivers of PL supply which we use to estimate the entry/exit models. We re-estimated the entry-exit models with changes in drivers from 2010 to 2012 (Table WD4) and the subsequent outcome models with changes from 2012 to 2017 (Table WD5) and found significant impacts (with some change in magnitude) of entry and exit on distribution depth but limited impacts on share outcomes.

Table WD1: Multivariate Probit Model Estimates for PL Supply

	Mercadona	Dia	Carrefour	Eroski	Alcampo	El Corte Ingles
$Growth_{m}$	$-.468^{**}$	-.105	-.076	$-.201^{**}$	$-.189^{*}$	.004
	(-2.25)	(-1.51)	(-1.00)	(-2.21)	(-1.71)	(.05)
$Sales_{mc}$	$.140^{*}$	$.089^{***}$	$.055^{*}$	$.140^{***}$	$.058^{**}$	$.062^{**}$
	(1.92)	(2.62)	(1.81)	(4.97)	(2.50)	(2.08)
$NewinCat_{mc}$	.373	-.146	-.183	-.219	-.257	-.144
	(1.04)	(-.82)	(-.86)	(-.83)	(-.90)	(-.73)
$NumCats_{m}$	.063	.042	-.041	-.004	.019	$.141^{*}$
	(.88)	(.49)	(-.74)	(-.07)	(.25)	(1.69)
$NBperCat_{m}$	-.042	-.042	.015	.024	.039	.010
	(-.43)	(-.60)	(.28)	(.43)	(.79)	(.18)
$PricePrem_{mc}$	.450	-.415	$.295^{*}$	-.222	.306	$-.806^{**}$
	(1.37)	(-.85)	(1.87)	(-.73)	(1.01)	(-1.97)
$NewSKUs_{mc}$	-.064	$.198^{***}$	$.136^{**}$	$.147^{**}$	.083	.082
	(-.67)	(4.03)	(2.28)	(1.99)	(1.59)	(1.28)
$Prem{+}Fighter_{mc}$	-.073	$.405^{**}$	$.270^{*}$	.143	.138	.187
	(-.48)	(2.49)	(1.86)	(1.02)	(1.07)	(1.48)
$Rshare_{rc}$	-.621	3.470^{***}	-.724	-.858	3.473	-9.888
	(-.61)	(3.40)	(-.66)	(-1.12)	(1.19)	(-1.40)
$PLShare_{rc}$	.444	.162	$.615^{**}$	$.567^{**}$	1.199^{***}	$.522^{**}$
	(1.07)	(.60)	(2.16)	(2.09)	(3.23)	(2.43)
$PLPriceRat_{rc}$	-.443	.084	.235	-.070	.519	.157
	(-.45)	(.21)	(.47)	(-.13)	(1.28)	(.27)
$CompIntensity_{mcr}$	1.396	.105	-.358	.336	1.349^{**}	$.851^{*}$
	(1.46)	(.20)	(-.67)	(.44)	(2.13)	(1.66)
$DistDepth_{mcr}$	-.555	-.413	$-.423^{**}$	$-.692^{***}$	-.207	$-.638^{*}$
	(-1.10)	(-1.11)	(-2.21)	(-2.85)	(-.81)	(-1.84)
$Share_{mcr}$	1.858^{*}	.182	.744	.726	.077	.801
	(1.93)	(.19)	(.94)	(.74)	(.07)	(1.28)

MdependonR$_{mr}$	2.035***	.936**	.259	1.667***	.859***	1.416***
	(5.00)	(2.30)	(1.00)	(4.30)	(3.10)	(4.64)
RDependonM$_{mr}$	-30.868	-41.445	-26.116**	-38.182*	-32.408**	-28.655
	(-.82)	(-1.45)	(-2.11)	(-1.70)	(-2.52)	(-1.16)
Beverage$_{c}$	.063	.341*	.041	-.355	-.089	-.155
	(.19)	(1.70)	(.20)	(-1.32)	(-.45)	(-.75)
PersonalCare$_{c}$	-.105	-.222	-.456	-.329	-.175	-.502*
	(-.35)	(-.91)	(-1.51)	(-1.17)	(-.59)	(-1.77)
HouseholdProd$_{c}$	.361	.297**	.216	.196	.321*	.053
	(1.04)	(2.32)	(1.35)	(.93)	(1.94)	(.32)
Copula Prem+Fighter$_{mc}$	.026	-.026	-.054	-.047	.042	-.028
	(.58)	(-.46)	(-1.30)	(-1.05)	(1.08)	(-.80)
Copula PricePrem$_{mc}$	-.369*	-.052	-.228*	.094	-.406**	.433*
	(-1.78)	(-.18)	(-1.78)	(.49)	(-2.25)	(1.79)
Copula PLPriceRat$_{rc}$	.232	-.090	-.008	.004	-.113	-.004
	(.53)	(-.66)	(-.05)	(.02)	(-.79)	(-.02)
Copula DistDepth$_{mcr}$	-.058	.002	-.098*	.036	-.123**	.102
	(-.59)	(.02)	(-1.73)	(.58)	(-2.09)	(1.05)
Copula Share$_{mcr}$	.039	.056	.291***	.098	.224***	.237***
	(.80)	(.86)	(3.51)	(1.27)	(2.98)	(3.52)
Constant	-2.446***	-2.186***	-1.727***	-1.964***	-1.680***	-2.713***
	(-7.80)	(-25.74)	(-27.13)	(-26.60)	(-6.75)	(-3.77)

Note: t statistics in parentheses. *** $p < .01$; ** $p < .05$; * $p < .10$; two-tailed test. N = 4,201.

Estimated MVP Covariance Matrix

	Mercadona	Dia	Carrefour	Eroski	Alcampo	El Corte Ingles
Mercadona	1.00[a]	.06	.04	.01	.08	.14
Dia		1.00	.60***	.43***	.48***	.39***
Carrefour			1.00	.47***	.55***	.39***
Eroski				1.00	.45***	.33***
Alcampo					1.00	.39***
El Corte Ingles						1.00

[a] all diagonal elements are normalized to 1. *** $p < .01$; ** $p < .05$; * $p < .10$; two-tailed test.

Table WD2: Meta-Analytic Zs for Independent vs. Multivariate Probit PL Supply Model

**Variable**	**Meta-Analytic Z from Probit Models**	**"Meta-Analytic" Z from Multivariate Probit Model**
Manufacturer Characteristics		
$Growth_{m}$	-3.25***	-3.52***
$Sales_{mc}$	7.99***	6.50***
$NewinCat_{mc}$	-3.59***	-1.27
$NumCats_{m}$	.25	1.02
$NBperCat_{m}$	1.40	.27
$PricePrem_{mc}$	-1.14	.29
$NewSKUs_{mc}$	5.23***	4.29***
$Prem+Fighter_{mc}$	2.66***	3.04***
Retailer Characteristics		
$RShare_{rc}$	-.25	.32
$PLShare_{rc}$	4.83***	4.73***
$PLPriceRat_{rc}$	.30	.67
Dyad Characteristics		
$CompIntensity_{mcr}$	3.46***	2.13**
$DistDepth_{mcr}$	-4.36***	-4.05***
$Share_{mcr}$	.69	2.11**
$MDependonR_{mr}$	9.38***	8.30***
$RDependonM_{mr}$	-3.48***	-3.99***

*** p < .01; ** p < .05; * p < .10; two-tailed test.

Table WD3: Meta-Analytic Zs with 2011 vs. 2010 PL Supply Drivers

**Variable**	**Meta-Analytic Z for Main Effect in NB Cats.**		**Meta-Analytic Z for Total Effect in Non-NB Cats.** [a]	
	2010 Drivers	2011 Drivers	2010 Drivers	2011 Drivers
Manufacturer Characteristics				
$NoNB_{mc}$	-13.77***	-9.33***		
$Growth_{m}$	-3.25***	-5.00***	-2.26***	-3.86***
$Sales_{mc}$	7.99***	8.99***	3.39***	2.72***
$NewinCat_{mc}$	-3.59***	-2.41**	.74	-.49
$NumCats_{m}$	.25	.29	5.61***	5.17***
$NBperCat_{m}$	1.40	.56	2.77***	2.91***
$PricePrem_{mc}$	-1.14	-2.82***	-.05	-1.49
$NewSKUs_{mc}$	5.23***	4.55***	-.92	.31
$Prem+Fighter_{mc}$	2.66***	2.34**	-.50	-.01
Retailer Characteristics				
$RShare_{rc}$	-.25	-.64	-1.34	-1.34
$PLShare_{rc}$	4.83***	4.48***	-.91	-1.06
$PLPriceRat_{rc}$	.30	.47	-.83	-.31
Dyad Characteristics				
$CompIntensity_{mcr}$	3.46***	2.98***	3.04***	2.10**
$DistDepth_{mcr}$	-4.36***	-2.77**	-2.76***	-1.96**
$Share_{mcr}$	.69	.11	-.21	-.97
$MDependonR_{mr}$	9.38***	9.13***	3.32***	3.19***
$RDependonM_{mr}$	-3.48***	-3.48***	-1.43	-.53

Note: The model also includes five copula terms and department fixed effects.

[a] Computed from the Z-statistics of the variable's total effect (main coefficient plus interaction with $NoNB_{mc}$).

*** $p < .01$; ** $p < .05$; * $p < .10$; two-tailed test.

Table WD4: Entry and Exit Models with 2010-2012 Changes in Drivers

Variable	PL Supply Entry Model[a]		PL Supply Exit Model[a]	
	Coefficient	t-stat	Coefficient	t-stat
Δ $Growth_{m}$	.03	.87	-.01	-.10
Δ $Sales_{mc}$	.04*	1.78	-.17***	-2.89
Δ $NumCat_{sm}$	.20	1.32	-.22	-1.05
Δ $NBperCat_{m}$	.05	.67	.15	1.64
Δ $PricePrem_{mc}$	.03	.43	.25*	1.72
Δ $NewSKUs_{mc}$	.04	1.06	-.10	-1.49
Δ $Prem{+}Fighter_{mc}$	-.05	-.60	-.18	-1.55
Δ $RShare_{rc}$	.48	.37	-.61	-.16
Δ $PLShare_{rc}$	-.10	-.46	-.69	-1.19
Δ $PLPriceRat_{rc}$	.12	.98	.04	.21
Δ $CompIntensity_{mcr}$	.59	1.28	-2.04**	-2.45
Δ $DistDepth_{mcr}$	.01	.11	-.27	-1.24
Δ $Share_{mcr}$	3.50***	5.33	-.23	-.25
Δ $MDependonR_{mr}$	.40	1.26	.19	.49
Δ $RDependonM_{mr}$	6.76	.23	-60.62	-1.08
$PLSupORetsSameCat_{mc}$	.78***	11.19	-.08	-.92
$PLSupOCats_{mc}$	.2***	5.02	-.08	-1.27
Mercadona	-.55***	-4.32	-.15	-.46
Dia	.29***	3.45	.26*	1.76
Carrefour	.16**	1.99	.14	.91
Eroski	.17**	2.03	-.09	-.63
El Corte Ingles	.04	.52	.15	1.03
Constant	-2.56***	-34.39	-.92***	-8.11
Pseudo R^2	.14		.05	
N	35,779		1,391	

[a] Pooled estimates across retailers because pooling was not rejected.

*** $p < .01$; ** $p < .05$; * $p < .10$; two-tailed test.

Table WD5: 2012-2017 Distribution Depth and Share Outcome Models

Variable	Impact of Entry on		Impact of Exit on	
	Δ Rel. Dist. Depth	Δ Rel. Share	Δ Rel. Dist. Depth	Δ Rel. Share
$PLentry_{mrc}$	.076**	-.031		
	(2.50)	(-1.23)		
$GenResidEntry_{mrc}$	-.024*	.012		
	(-1.82)	(1.27)		
$PLexit_{mrc}$			-.220***	-.030*
			(-3.89)	(-1.74)
$GenResidExit_{mrc}$			.115***	.016
			(3.34)	(1.59)
Δ $RShare_{rc}$	.060	-.007	.042	-.268*
	(1.22)	(-.66)	(.02)	(-1.73)
Δ $PLShare_{rc}$	.006	.002	.028	-.080**
	(.32)	(.93)	(.29)	(-2.12)
Δ $PLPriceRat_{rc}$	-.007	-.002	.003	-.091**
	(-1.12)	(-.91)	(.13)	(-2.45)
Δ $CompIntensity_{mcr}$	.045	-.002	-.061	-.023
	(.62)	(-.35)	(-.56)	(-.64)
Δ $DistDepth_{mcr}$	-.359***	.002*	-.427***	.003
	(-43.53)	(1.90)	(-13.20)	(.40)
Δ $Share_{mcr}$	.163**	-.286***	.281**	-.551**
	(3.13)	(-6.12)	(1.98)	(-2.26)
Δ $MDependonR_{mr}$	.017	-.005	.083	.003
	(.28)	(-1.04)	(.83)	(.23)
Δ $RDependonM_{mr}$	-2.263	.232	3.606	2.508
	(-.57)	(.25)	(.25)	(.74)
Mercadona	.044***	.001	.034	.002
	(9.09)	(1.22)	(.92)	(.19)
Dia	.047***	.003***	.036	.005
	(8.56)	(4.34)	(1.57)	(1.01)

Carrefour	.047***	.002***	.062*	.009
	(8.30)	(3.33)	(1.88)	(1.20)
Eroski	.016**	.003***	.061**	.011
	(2.38)	(4.46)	(2.36)	(1.28)
El Corte Ingles	-.001	.001	-.034	.008
	(-.08)	(1.22)	(-1.42)	(1.40)
Mercadona x Δ $RShare_{rc}$			-1.004	
			(-.34)	
Dia x Δ $RShare_{rc}$			1.166	
			(.47)	
Carrefour x Δ $RShare_{rc}$			-1.161	
			(-.41)	
Eroski x Δ $RShare_{rc}$			2.809	
			(.99)	
El Corte Ingles x Δ $RShare_{rc}$			5.083***	
			(2.87)	
Mercadona x Δ $PLShare_{rc}$				.008
				(.04)
Dia x Δ $PLShare_{rc}$				.095*
				(1.69)
Carrefour x Δ $PLShare_{rc}$				.131**
				(2.02)
Eroski x Δ $PLShare_{rc}$				.153***
				(2.52)
El Corte Ingles x Δ $PLShare_{rc}$				.041
				(.67)
Mercadona x Δ $PLPriceRat_{rc}$		.002		.108**
		(.65)		(2.08)
Dia x Δ $PLPriceRat_{rc}$		-.003		.108***
		(-1.10)		(2.87)

Carrefour x Δ $PLPriceRat_{rc}$		$.006^{**}$		$.140^{**}$
		(2.17)		(2.30)
Eroski x Δ $PLPriceRat_{rc}$		.005		$.089^{*}$
		(1.16)		(1.92)
El Corte Ingles x Δ $PLPriceRat_{rc}$		$.006^{*}$		$.090^{**}$
		(1.62)		(2.45)
Mercadona x Δ $Share_{mcr}$				.119
				(.34)
Dia x Δ $Share_{mcr}$				.028
				(.07)
Carrefour x Δ $Share_{mcr}$				$.747^{***}$
				(2.74)
Eroski x Δ $Share_{mcr}$				.181
				(.50)
El Corte Ingles x Δ $Share_{mcr}$				.139
				(.60)
Mercadona x Δ $CompIntensity_{mcr}$	-.070			
	(-.74)			
Dia x Δ $CompIntensity_{mcr}$	$-.202^{***}$			
	(-2.75)			
Carrefour x Δ $CompIntensity_{mcr}$	$-.214^{**}$			
	(-2.35)			
Eroski x Δ $CompIntensity_{mcr}$	-.086			
	(-.88)			
El Corte Ingles x Δ $CompIntensity_{mcr}$	.097			
	(1.06)			
Mercadona x Δ $MDependonR_{mr}$	.114	.002		
	(1.51)	(.65)		
Dia x Δ $MDependonR_{mr}$	-.007	-.003		
	(-.05)	(-1.10)		
Carrefour x Δ $MDependonR_{mr}$	$.175^{**}$	$.006^{**}$		

	(2.00)	(2.17)		
Eroski x Δ $MDependonR_{mr}$	.117	.005		
	(1.13)	(1.16)		
El Corte Ingles x Δ $MDependonR_{mr}$	.097	.006		
	(1.06)	(1.62)		
Constant	-.035***	-.000	.020	.003
	(-7.57)	(-.07)	(.32)	(.53)
Adjusted R^2	.12	.05	.13	.14
N	35,779	35,779	1,391	1,391

Note: In each model, retailer-specific coefficients are specified only for those variables where pooling is rejected.
t statistics in parentheses. * $p < .1$, ** $p < .05$, *** $p < .01$
